# Vitamin D Promotes Ferroptosis in Colorectal Cancer Stem Cells via SLC7A11 Downregulation

**DOI:** 10.1155/2023/4772134

**Published:** 2023-02-16

**Authors:** Shuang Guo, Wei Zhao, Weihao Zhang, Shuai Li, Guoxin Teng, Lan Liu

**Affiliations:** ^1^Department of Gastroenterology, The Second Hospital of Shandong University, Jinan, Shandong 250033, China; ^2^Department of Pathology, The Second Hospital of Shandong University, Jinan, Shandong 250033, China

## Abstract

Colorectal cancer stem cells (CCSCs) play important roles in the prognosis, chemoresistance, and treatment failure of colorectal cancer (CRC). Ferroptosis is an effective treatment for CCSCs. Vitamin D (VD) reportedly inhibits colon cancer cell proliferation. However, information on the relationship between VD and ferroptosis in CCSCs is not well documented. In this study, we aimed to understand the effect of VD on ferroptosis in CCSCs. To this end, we treated CCSCs with different concentrations of VD and performed spheroid formation assay and transmission electron microscopy and determined cysteine (Cys), glutathione (GSH), and reactive oxygen species (ROS) levels. Furthermore, functional experiments, western blotting, and qRT-PCR were performed to explore the downstream molecular mechanisms of VD *in vitro* and *in vivo*. Results showed that VD treatment significantly inhibited the proliferation of CCSCs and reduced the number of tumour spheroids *in vitro*. Further evaluations showed that the VD-treated CCSCs exhibited significantly higher ROS levels and lower levels of Cys and GSH as well as thickened mitochondrial membranes. Furthermore, the mitochondria in CCSCs were narrowed and ruptured after VD treatment. These results indicated that VD treatment significantly induced ferroptosis in CCSCs. Further exploration showed that SLC7A11 overexpression significantly attenuated VD-induced ferroptosis *in vitro* and *in vivo*. Hence, we concluded that VD induces ferroptosis in CCSCs by downregulating SLC7A11 *in vitro* and *in vivo*. These results provide new evidence for the therapeutic use of VD in treating CRC and new insights into VD-induced ferroptosis in CCSCs.

## 1. Introduction

Colorectal cancer (CRC) is a common malignant cancer with high incidence and mortality rates [1]. Surgery is the basic treatment for CRC, while adjuvant chemotherapy is routinely used to improve patient outcomes [2]. Recent studies have shown that colorectal cancer stem cells (CCSCs) induce multistep and multistage characteristics, including chemotherapy failure and tumour recurrence, during the pathological process of CRC [3]. Thus, targeting CCSCs could improve the therapeutic effect of CRC [4].

Ferroptosis, a form of nonapoptotic cell death discovered in 2012, is dependent on intracellular iron levels and the accumulation of lipid reactive oxygen species (ROS) in the cell [5]. ROS production and aberrant lipid and iron metabolism are some of the physiological differences between cancer and normal cells [6]. Since these characteristics play essential roles in regulating ferroptosis, cancer cells could be more susceptible to modulations in this death pathway compared to normal cells [7]. Some data demonstrate that ferroptosis inducers can specifically target cancer stem cells (CSCs) in the tumour [8]. Because of their more aberrant lipid metabolism and ROS production, CSCs are specifically susceptible to ferroptosis [9]. Therefore, ferroptosis may play an important role in achieving complete tumour eradication and overcoming chemotherapy resistance, as it has superior selectivity and efficacy in inducing CSC death [8]. Solute carrier family 7 member 11 (SLC7A11)/system xc− inhibition (e.g., sorafenib, erastin, and sulfasalazine), glutathione peroxidase 4 (GPX4) inhibition (e.g., Ras-selective lethal compound 3 (RSL3) and FIN56), and physiological conditions (e.g., high extracellular glutamate and cystine deprivation) are all reported to trigger ferroptosis [10, 11]. The increased expression of cyclooxygenase 2 (COX2) is also a hallmark of ferroptosis [12]. There is also evidence demonstrating that ferroptosis is involved in CRC activities. For instance, RSL3 promotes cell death and accumulation of ROS and induces ferroptosis in CRC cells [13]. Xu et al. reported that ferroptosis can specifically kill CCSCs via downregulating SLC7A11, thus attenuating chemotherapy resistance in CRC [14]. However, further research is required on the relationship between ferroptosis and CCSCs and the underlying mechanism.

Vitamin D (VD) has potential applications in cancer therapy. The active form of VD, 1*α*,25-(OH)_2_D_3_, inhibits tumour growth [15]; additionally, through binding to the VD receptor (VDR), it plays a transcriptional regulatory role by facilitating the combination of its DNA-binding domain with VD response elements in target genes [16, 17]. Studies have shown that VD and VDR affect the stemness of CCSCs and that the acidic tumour microenvironment leads to a reduction in VDR expression and affects the stemness of CRC cells by regulating the expression of SOX2 [18]. Furthermore, VD reportedly regulates iron metabolism and the hepcidin–ferroportin axis in humans and mammals [19]. Moreover, dietary VD affects the iron ion transport signaling pathway in the immune tissues of yellow catfish, as evidenced by next-generation sequencing results [20]. However, information on the relationship between VD and ferroptosis in CCSCs is not well documented.

Therefore, we aimed to determine the relevance of VD and ferroptosis in CCSCs and explore the downstream molecular mechanisms of ferroptosis in CCSCs treated with VD, both *in vitro* and *in vivo*.

## 2. Materials and Methods

### 2.1. Isolation of Primary CRC Cells and CCSCs

Tumour tissues were obtained from 20 patients with CRC who underwent surgery at the Second Hospital of Shandong University between April 2018 and July 2020. Primary CCSCs were isolated from fresh tissue samples. The samples were minced and digested in collagenase I and trypsin (Gibco, Grand Island, NY, USA) at 37°C for 1 h. Then, a 40 *μ*m cell strainer was used to filter the digested tissues. Cells were cultured in Dulbecco's modified Eagle's medium (DMEM)/F-12 medium (MACGENE, Beijing, China) supplemented with 20 ng/mL epidermal growth factor (EGF) (PeproTech, Rocky Hill, NJ, USA), 20 ng/mL basic fibroblast growth factor (bFGF) (PeproTech), 2% B27 (Invitrogen, Carlsbad, CA, USA), and 10 *μ*g/mL heparin (Invitrogen). Then, the stem cell suspension was isolated for detection. The human CRC cell line HCT-116 was purchased from the American Type Culture Collection (CCL-247; ATCC, Rockefeller, MA, USA).

### 2.2. Spheroid Formation Assay

Briefly, 1 × 10^6^ CCSCs were cultured in DMEM/F-12 (MACGENE), containing 20 ng/mL EGF, 20 ng/mL bFGF, 2% B27, and 10 *μ*g/mL heparin, in ultralow-attachment six-well plates (Corning, Corning, NY, USA). All the plates were covered with 95% poly (2-hydroxyethyl methacrylate) (Sigma-Aldrich, St. Louis, MO, USA). After two weeks of cultivation, spheroids of size > 80 *μ*m were observed and counted using an inverted fluorescence microscope (CKX53, Olympus, Tokyo, Japan).

### 2.3. Cell Proliferation Analysis

The VD metabolite 1,25D3 (Sigma-Aldrich) was stored in anhydrous alcohol (AA) at a concentration of 400 *μ*M and was diluted to final concentrations of 50 nmol/L (nM) and 100 nM prior to use. The cells were seeded at a density of 3 × 10^4^ cells/well in a 96-well plate and incubated for 24 h. Different concentrations of VD (0, 50, and 100 nM) were added to the wells. From 24 to 72 h after incubation, cell proliferation analysis was performed using Cell Counting Kit-8 (CCK-8) (Dojindo, Kumamoto, Japan) according to the manufacturer's instructions.

### 2.4. Flow Cytometric Analysis

To detect the presence of CD133^+^ and CD44^+^ cells, anti-human/mouse CD44 (F1104401, 1 : 20, MULTISCIENCES, Hangzhou, China), fluorescein isothiocyanate (FITC) (MULTISCIENCES), and FITC anti-human CD133 antibody (567908, 1 : 20, BD Biosciences, Franklin Lakes, NJ, USA) were used to label the cells. Then, the cells were detected via flow cytometry (CytoFLEX, BECKMAN, Brea, CA, USA). Furthermore, CCSCs were treated with 1 *μ*M oxaliplatin (#HY-17371, MedChemExpress, Monmouth Junction, NJ, USA) and 100 nM VD. A living/dead cell double staining assay was performed using the Calcein-AM/PI Kit (Solarbio, Beijing, China), according to the manufacturer's instructions. Dead cells were stained with PI, and their percentages were revealed when detected using flow cytometry.

### 2.5. Evaluation of Cys, GSH, and ROS Levels

The Cys concentration and GSH levels in cells were determined using the Cys Content Assay Kit (Solarbio) and GSH Detection Kit (Solarbio), respectively, according to the manufacturer's instructions. A Reactive Oxygen Species Assay Kit (S0033S, Beyotime, Shanghai, China) was used to detect ROS levels via flow cytometry; the excitation and emission wavelengths were 488 and 525 nm, respectively.

### 2.6. Quantitative Reverse Transcription-Polymerase Chain Reaction (qRT-PCR)

Total RNA was extracted from cells using TRNzol Universal (Transgen, Beijing, China), and complementary DNA (cDNA) was synthesised using the FastQuant First-Strand cDNA Synthesis Kit (Transgen). Then, qRT-PCR was performed using a real-time fluorescence quantitative PCR device (LightCycler96, Basel, Switzerland). The relative expression levels of transcripts were calculated using the 2^-△△Ct^ method. *β*-Actin served as an internal reference. The primer sequences used were as follows: SOX2 forward, 5′-AACCAGCGCATGGACAGTTA-3′, SOX2 reverse, 5′-GACTTGACCACCGAACCCAT-3′; OCT4 forward, 5′-GCTGGATGTCAGGGCTCTTT-3′, OCT4 reverse, 5′-AACCACACTCGGACCACATC-3′; Nanog forward, 5′-AATGGTGTGACGCAGGGATG-3′, Nanog reverse, 5′-TGCACCAGGTCTGAGTGTTC-3′; SLC7A11 forward, 5′-GGTCAGAAAGCCTGTTGT-3′, SLC7A11 reverse, 5′-GTTCCACCCAGACTCGTA-3′; GPX4 forward, 5′-CCTTTGCCGCCTACTGAA-3′, GPX4 reverse, 5′-ACTCCCTGGCTCCTGCTT-3′; COX2 forward, 5′-GCCTTTGCCGCCTACTGA-3′, COX2 reverse, 5′-CGTTACTCCCTGGCTCCTG-3′; and *β*-actin forward, 5′-GGTAATGGTAGGTATGGGACA-3′, *β*-actin reverse, 5′-TCTTCAGGAGCAACACGA-3′.

### 2.7. Western Blotting

Cells were lysed in a Cell Lysis Buffer (Sigma-Aldrich) and denatured by heating at 95°C for 15 min with the loading buffer (BioTNT, Shanghai, China). Approximately 20 *μ*g of total protein was separated through sodium dodecyl sulphate-polyacrylamide gel electrophoresis and transferred onto polyvinylidene fluoride membranes (Millipore, Darmstadt, Germany). The membranes were incubated with primary and secondary antibodies, and a chemiluminescence assay was performed using ECL western blot assay reagent (Pierce, Itasca, IL, USA). The primary antibodies used were as follows: SRY-box transcription factor 2 (SOX2) (2748S, 1 : 1000, Cell Signaling, Boston, MA, USA), organic cation/carnitine transporter4 (OCT4) (2750S, 1 : 1000, Cell Signaling), Nanog homeobox (Nanog) (3580S, 1 : 1000, Cell Signaling), SLC7A11 (26864-1-AP/R, 1 : 1000, Proteintech, Wuhan, China), glutathione peroxidase 4 (GPX4) (14432-1-AP/R, 1 : 1000, Proteintech), cytochrome c oxidase subunit II (COX2) (66351-1-Ig/M, 1 : 1000, Proteintech), and *β*-actin (TA-09/M, 1 : 5000, ZSGB-BIO, Beijing, China). The gray value of protein expression was analysed using the ImageJ software. The relative protein expression levels were normalised to those of *β*-actin.

### 2.8. Lentivirus-Mediated Overexpression and Knockdown

The shRNA sequences against human SLC7A11 were designed and inserted into a modified pLV-H1-Puro lentiviral vector. The corresponding sequences for sh-SLC7A11 were 5′-GCTTTGTCTTATGCTGAATTG-3′ (shRNA-1), 5′-GCAGCTAATTAAAGGTCAAAC-3′ (shRNA-2), and 5′-GGGCTGATTTATCTTCGATAC-3′ (shRNA-3). The human SLC7A11 was amplified via reverse transcription PCR and cloned into a modified pLV-EF1*α* lentiviral vector. For infection, CCSCs were incubated with lentiviruses (multiplicity of infection (MOI) of 10) in a serum-free medium in a 6-well plate. After 48 h of transfection, the infected cells were selected using puromycin (2 mg/mL) and transferred to a culture medium containing serum for 48 h. qRT-PCR was performed to determine the efficiency of inhibition and overexpression.

### 2.9. *In Vivo* Tumour Model

We purchased 5-week-old male BALB/c nude mice from the Experimental Animal Center of Shandong University, China. SLC7A11 was knocked out or overexpressed in CCSCs. Subsequently, the cells were washed thrice with cold phosphate-buffered saline (PBS), and 1 × 10^6^ cells were subcutaneously injected into the backs of nude mice. VD was injected intraperitoneally at a concentration of 30 g/kg once every two days. The mice were randomly divided into six groups—control, VD, sh-SLC7A11, VD+sh-SLC7A11, overexpressed (OE)-SLC7A11, and VD+OE-SLC7A11 groups (*n* = 5 mice in each group). The volume of tumours was calculated using the formula *V* = *L* (the long diameter) × *W*^2^ (the short diameter) × 0.5 for caliper measurement every week. Finally, the mice were euthanised after five weeks, and tumours were excised and weighed. Then, we evaluated Cys, GSH, and ROS levels as well as GPX4, COX2, and SLC7A11 protein levels in the tumour tissues.

### 2.10. Immunofluorescence Staining

The cells were fixed using 4% paraformaldehyde for 15 min and permeabilised with 0.2% Triton X-100 for 20 min at 20°C. Then, the cells were blocked with 10% goat serum and incubated with Ki67 polyclonal antibody (27309-1-AP, 1 : 500, Proteintech) overnight at 4°C. After washing with PBS, an anti-rabbit antibody (4412, 1 : 1000, Cell Signaling) was added. After incubating for 1 h at 20°C, the cells were stained with 4′,6-diamidino-2-phenylindole (Solarbio) to visualise the nuclei. All images were captured using a fluorescence microscope (ECLIPSE Ci, NIKON, Tokyo, Japan); representative images are shown. Then, the fluorescent intensity was quantitated from the micrographs using the ImageJ software.

### 2.11. Transmission Electron Microscopy

Cells were seeded at a density of 2 × 10^5^ cells per well in a six-well plate and cultured continuously for 12 h. Different intervention reagents were used, and cells were further cultured for 48 h. Then, the cells were harvested and fixed with 2% glutaraldehyde. Finally, the samples were observed under an electron microscope (JSM-6610LV, Tokyo, Japan) at Shandong University.

### 2.12. Bioinformatic Analysis

The mRNA sequencing (RNA-seq) data of 194 samples from colon cancer patients and 41 adjacent tissues samples were downloaded from TCGA website (https://portal.gdc.cancer). The R software (Version 3.3.6; The R Foundation for Statistical Computing, Vienna, Austria) and Bioconductor packages (impute and limma) were used to analyse differentially expressed genes (DEGs). The threshold was set to log_2_ (fold change (FC)) > 2 and *P* value < 0.05. Then, we obtained ferroptosis-related prognostic genes (FRGs) from the FerrDb online database (http://www.zhounan.org/ferrdb/) [21] and took the intersection of DEGs and the FRGs to obtain differentially expressed ferroptosis-related genes. We also used GEPIA (http://gepia.cancer-pku.cn) to perform differential expression analysis of candidate proteins.

### 2.13. Statistical Analysis

Data analysis was performed using SPSS 17.0 (SPSS Inc., Chicago, IL, USA) and GraphPad Prism 6 (GraphPad, La Jolla, CA, USA). The significance of differences was determined through a one-way analysis of variance (ANOVA) or Student's *t*-test. All data are presented as mean ± standard deviation. If there were more than three groups, ANOVA followed by Tukey's post hoc test was used. *P* < 0.05 was considered as significant.

## 3. Results

### 3.1. Isolation and Identification of CCSCs

**Figure 1:**
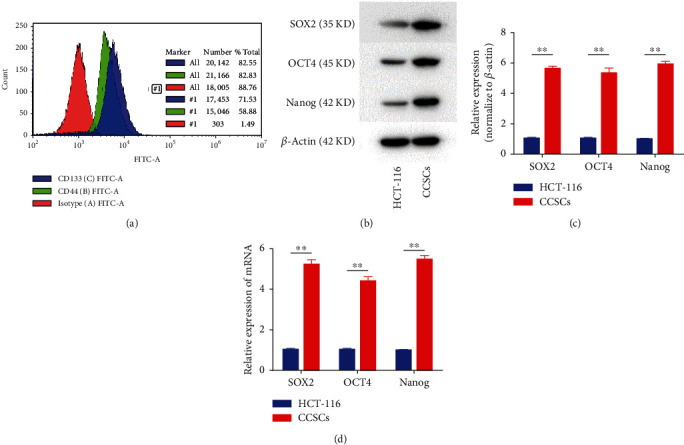
Phenotype identification of colorectal cancer stem cells (CCSCs). (a) Phenotypes of suspension stem cells. Count and FITC-A are axis labels, #1 is gate label, and there were no quartile labels. (b, c) Western blotting and quantitative analysis of stemness markers, including SOX2, OCT4, and Nanog, in CCSCs. (d) qRT-PCR analysis of stemness markers, including SOX2, OCT4, and Nanog, in CCSCs. Data are presented as mean ± SD; ^∗∗^*P* < 0.01.

Cell surface markers in the isolated spheroids were detected using flow cytometry. Results showed that the percentages of CD133^+^ and CD44^+^ cells in the stem cell suspension were 71.53% and 58.88%, respectively (Figure 1(a)). The expression levels of stemness markers, including SOX2, OCT4, and Nanog, in the stem cell suspension were increased compared with those in the HCT-116 cells (Figures 1(b)–1(d)). Hence, we isolated the stem cell suspension as CCSCs for further study.

### 3.2. VD Inhibited Self-Renewability and Improved Chemosensitivity in CCSCs

**Figure 2:**
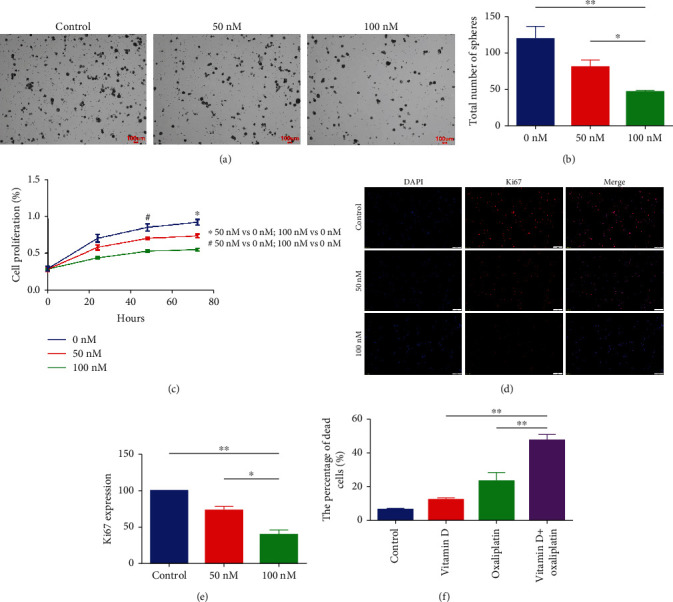
Vitamin D (VD) inhibited self-renewability and improved chemosensitivity in colorectal cancer stem cells (CCSCs). (a) Tumour spheroid assay of CCSCs treated with different concentrations of VD. Scale bars: 100 *μ*m. (b) Total number of tumour spheroids when CCSCs were treated with different concentrations of VD. (c) Proliferation ability of CCSCs treated with different concentrations of VD, assessed using the CCK-8 assay. (d, e) Immunofluorescence staining of CCSCs treated with varying concentrations of VD. Scale bars: 50 *μ*m. Representative photographs and quantitative analysis are shown. (f) Percentage of dead cells detected using flow cytometry after treating CCSCs with 1 *μ*M oxaliplatin and 100 nM VD. Data are presented as mean ± SD; ^∗^*P* < 0.05 and ^∗∗^*P* < 0.01.

To evaluate the effect of VD on the self-renewal of CCSCs, a tumour spheroid assay was employed. VD treatment significantly decreased the size and number of tumour spheroids formed by the CCSCs (Figures 2(a) and 2(b)). The CCK-8 assay was used to evaluate the effect of VD-mediated inhibition of CCSC proliferation; results showed that the proliferation of CCSCs in the VD group (100 nM) was significantly reduced (Figure 2(c)). Immunofluorescence staining of CCSCs illustrated a decrease in Ki67 expression in the group treated with 100 nm VD compared with that in the control (Figure 2(d)); quantitative analysis showed a statistically significant reduction (Figure 2(e)). In addition, when the CCSCs were treated with 1 *μ*M oxaliplatin and 100 nM VD, we found that VD treatment improved the drug sensitivity of oxaliplatin in CCSCs (Figure 2(f), Additional file 1).

### 3.3. VD-Induced Ferroptosis in CCSCs

**Figure 3:**
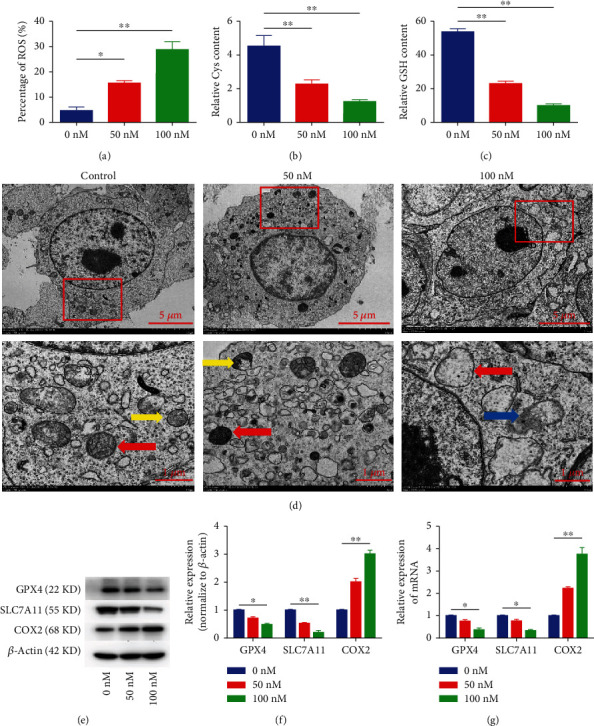
VD-induced ferroptosis in CCSCs. (a) Levels of ROS in the CCSCs treated with different concentrations of VD, as detected using flow cytometry. Results are expressed as percentages; bar graph summarises quantitative data representing the mean ± SD of values from three independent experiments. (b) Levels of Cys in the CCSCs treated with different concentrations of VD. (c) Levels of GSH in the CCSCs treated with different concentrations of VD. (d) Transmission electron microscopy of CCSCs treated with different concentrations of VD. The images below show an enlarged version of the red area. The red arrows indicate the thickening of the mitochondrial membrane and the narrowing of the mitochondria. The yellow arrows indicate the mitochondrial crista. The blue arrow indicates the ruptured mitochondria. Scale bars of the picture above: 5 *μ*m; scale bars of the picture below: 1 *μ*m. (e, f) Western blotting and quantitative analysis of GPX4, SLC7A11, and COX2 in CCSCs treated with different VD concentrations. (g) qRT-PCR analysis of GPX4, SLC7A11, and COX2 in CCSCs treated with different concentrations of VD. Data are presented as mean ± SD; ^∗^*P* < 0.05 and ^∗∗^*P* < 0.01.

To investigate whether ferroptosis is involved in the activity of CCSCs, we examined the levels of Cys, GSH, and ROS, which are the key executors of ferroptosis. We found that the cells in the VD group (100 nM) exhibited significantly higher ROS levels (Figure 3(a), Additional file 2) and lower Cys and GSH levels compared with that in the control (Figures 3(b) and 3(c)). Transmission electron microscopy results showed that the mitochondrial membrane was thickened and that mitochondria were narrowed and even ruptured in the CCSCs after VD treatment (Figure 3(d)), while COX2 was highly expressed and SLC7A11 and GPX4 levels were significantly downregulated in the VD group (Figures 3(e)–3(g)). These results demonstrated that VD treatment induced ferroptosis *in vitro* in CCSCs.

### 3.4. VD May Induce Ferroptosis through Downregulation of SLC7A11

**Figure 4:**
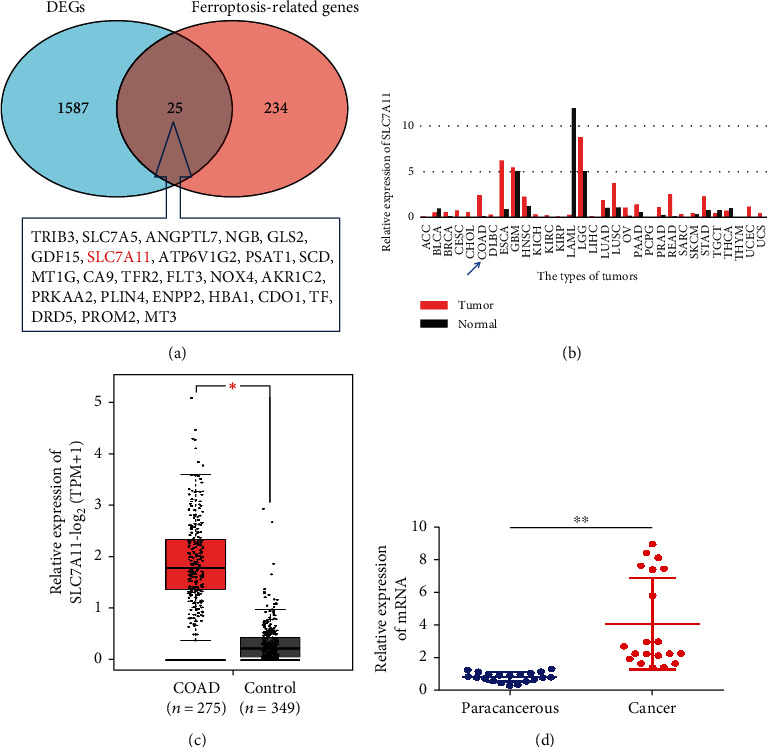
SLC7A11 may play an important role in the ferroptosis of colon cancer cells. (a) Venn diagram of colon cancer-related ferroptosis genes by the intersection of differentially expressed genes and ferroptosis-related prognostic genes. (b) Differential expression analysis of SLC7A11 in different types of tumours performed in the GEPIA database; the blue arrow indicates COAD. (c) Differential expression analysis of SLC7A11 in COAD performed in the GEPIA database. (d) qRT-PCR analysis of SLC7A11 expression in the colon cancer tissues and paracancer tissues. Data are presented as mean ± SD; ^∗^*P* < 0.05 and ^∗∗^*P* < 0.01.

We collected the data of colon cancer patients from TCGA database and analysed the DEGs in the colon cancer tissues. We identified 1587 DEGs and obtained 25 colon cancer-related ferroptosis genes by the intersection of DEGs and FRGs. SLC7A11 was included in the 25 genes, and its expression was increased, but GPX4 was not included (Figure 4(a)). Next, the GEPIA database was used to perform differential expression analysis of SLC7A11; we found that the expression of SLC7A11 was increased in colon adenocarcinoma (COAD), esophageal carcinoma (ESCA), lung squamous cell carcinoma (LUSC), and rectum adenocarcinoma (READ) and reduced in acute myeloid leukemia (LAML) (Figures 4(b) and 4(c)). Finally, we collected 20 pairs of colon cancer and paracancer tissues and observed a high expression of SLC7A11 in colon cancer tissues (Figure 4(d)). All these findings indicate that SLC7A11 plays an important role in the ferroptosis of colon cancer cells.

According to our previous results, SLC7A11 was significantly reduced after VD intervention in CCSCs. Therefore, we hypothesised that VD downregulated SLC7A11, inhibited Cys transport, reduced GSH synthesis, triggered ROS accumulation, and induced ferroptosis *in vitro* in CCSCs.

### 3.5. SLC7A11 Overexpression Improved the Proliferation and Inhibited Ferroptosis of CCSCs

**Figure 5:**
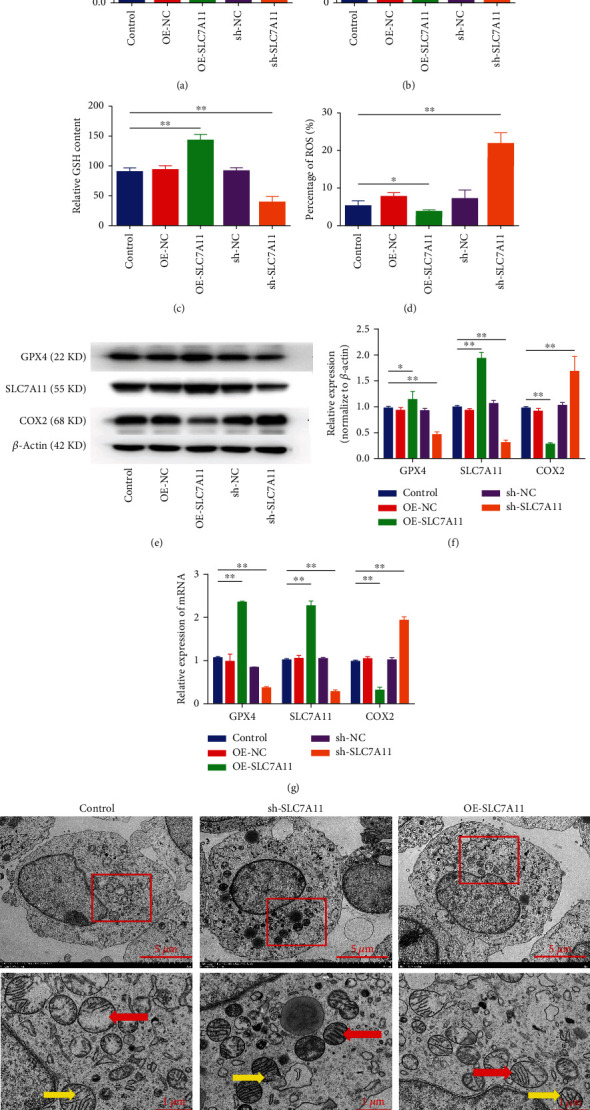
SLC7A11 overexpression improved the proliferation and inhibited ferroptosis of CCSCs. (a) Proliferative ability of CCSCs transfected with sh-SLC7A11 or OE-SLC7A11 as assessed using the CCK-8 assay. (b) Levels of Cys in CCSCs transfected with sh-SLC7A11 or OE-SLC7A11. (c) Levels of GSH in CCSCs transfected with sh-SLC7A11 or OE-SLC7A11. (d) Cellular levels of ROS in CCSCs transfected with sh-SLC7A11 or OE-SLC7A11, as detected using flow cytometry. Results are expressed as percentages; bar graph summarises the quantitative data as mean ± SD of values from three independent experiments. (e, f) Western blotting and quantitative analysis of GPX4, SLC7A11, and COX2 in CCSCs transfected with sh-SLC7A11 or OE-SLC7A11. (g) qRT-PCR analysis of GPX4, SLC7A11, and COX2 in CCSCs transfected with sh-SLC7A11 or OE-SLC7A11. (h) Transmission electron microscopy of CCSCs transfected with sh-SLC7A11 or OE-SLC7A11. The images below are enlarged version of the red area. The red arrows indicate thickening of the mitochondrial membrane and narrowing of the mitochondria. The yellow arrows indicate the mitochondrial crista. Scale bars of the picture above: 5 *μ*m; scale bars of the picture below: 1 *μ*m. Data are presented as mean ± SD; ^∗^*P* < 0.05 and ^∗∗^*P* < 0.01.

To examine the role of SLC7A11 in the ferroptosis of CCSCs, we knocked down SLC7A11 using a specific shRNA and overexpressed SLC7A11 transcripts via lentivirus transfection. We designed three shRNAs targeting SLC7A11, determined their efficacy using qRT-PCR, and finally selected the most efficient shRNA (sh#2) for subsequent experiments (Additional file 3A, B). The CCK-8 assay was used to evaluate the effect of SLC7A11 on CCSC proliferation. SLC7A11 overexpression significantly promoted the proliferation of CCSCs, increased Cys and GSH levels, and reduced ROS levels (Figures 5(a)–5(d), Additional file 3C). Several ferroptosis markers were assessed with regard to their mRNA and protein levels; SLC7A11 overexpression was found to significantly increase the protein and mRNA expression levels of GPX4 while suppressing those of COX2 (Figures 5(e)–5(g)). In contrast, knockdown of SLC7A11 significantly inhibited the proliferation of CCSCs, reduced Cys and GSH levels, and increased ROS levels. At the same time, knockdown of SLC7A11 significantly increased the protein and mRNA expression levels of COX2 while suppressing GPX4 levels. Transmission electron microscopy results demonstrated that the mitochondrial membrane was thickened and mitochondria were narrowed in cells in the sh-SLC7A11 group (Figure 5(h)). These findings indicate that SLC7A11 plays a critical role in the ferroptosis of CCSCs.

### 3.6. SLC7A11 Overexpression Rescued VD-Induced Ferroptosis in CCSCs

**Figure 6:**
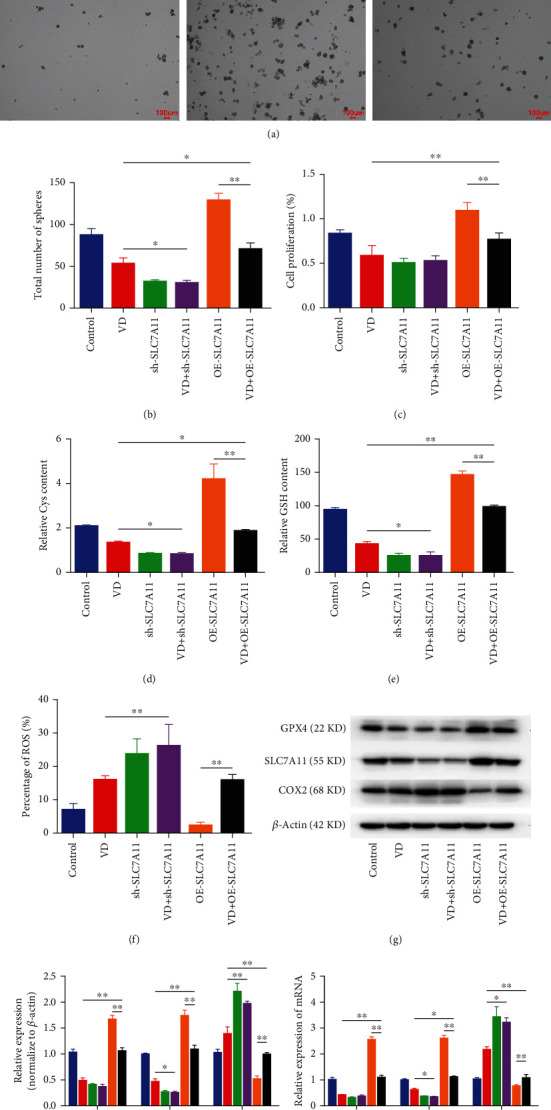
SLC7A11 overexpression rescued VD-induced ferroptosis in CCSCs *in vitro*. (a) Formation of spheroids in CCSCs of different groups. Scale bars: 100 *μ*m. (b) Number of tumour spheroids of CCSCs in different groups. (c) Proliferation ability of CCSCs in different groups, as assessed using the CCK-8 assay. (d) Levels of Cys in CCSCs of different groups. (e) Levels of GSH in CCSCs of different groups. (f) Cellular levels of ROS in CCSCs, as detected using flow cytometry. Results were expressed as percentages; bar graph summarises the quantitative data as mean ± SD of values from three independent experiments. (g, h) Western blotting and quantitative analysis of GPX4, SLC7A11, and COX2 in CCSCs of different groups. (i) qRT-PCR analysis of GPX4, SLC7A11, and COX2 in CCSCs of different groups. Data are presented as mean ± SD; ^∗^*P* < 0.05 and ^∗∗^*P* < 0.01.

To examine the role of SLC7A11 in VD-induced ferroptosis of CCSCs, we used lentivirus infection to overexpress SLC7A11 transcripts and shRNA (sh#2) to silence SLC7A11 and then performed VD intervention concomitantly. Results showed that the size and number of tumour spheroids were reduced after VD treatment, but overexpression of SLC7A11 alleviated this inhibition upon performing VD intervention and overexpression of SLC7A11 simultaneously (Figures 6(a) and 6(b)). The overexpression of SLC7A11 significantly rescued the reduction in Cys, GSH, and cell proliferation levels induced by VD (Figures 6(c)–6(e)). Regarding the levels of ferroptosis markers, the overexpression of SLC7A11 alleviated the inhibiting effect on the expression of GPX4 and the promoting effect on the expression of COX2 induced by VD (Figures 6(g)–6(i)). In contrast, knockdown of SLC7A11 significantly enhanced the effect of VD on the size and number of tumour spheroids, Cys and GSH levels, ROS levels, and the levels of ferroptosis markers, such as SLC7A11 and COX2 (Figures 6(a), 6(b), and 6(d)–6(i), Additional file 4). But after SLC7A11 silencing, VD had no significant promoting effect on the ferroptosis of CCSCs.

**Figure 7:**
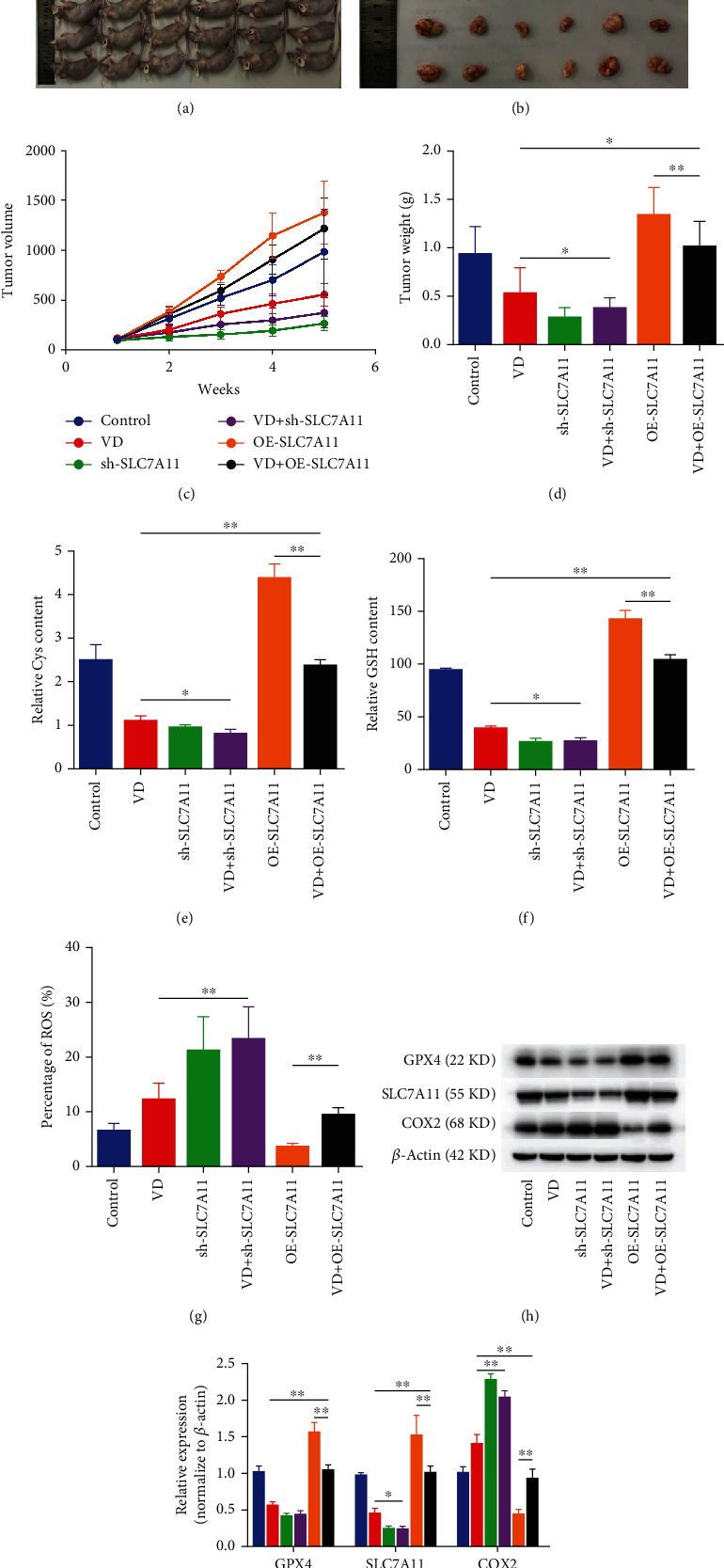
SLC7A11 overexpression rescued VD-induced ferroptosis in CCSCs *in vivo*. (a) Representative photographs of tumour-bearing nude mice. (b) Representative photographs showing excised tumours. (c) Changes in tumour volume in mice during the experimental period. (d) Weight of tumour excised from the mice in different groups after five weeks. (e) Levels of Cys in the tumours excised from nude mice. (f) Levels of GSH in the tumours excised from nude mice. (g) Cellular levels of ROS in the tumours excised from nude mice, as detected using flow cytometry. Results are expressed as percentages; bar graph summarises the quantitative data as mean ± SD of values from three independent experiments. (h, i) Western blotting and quantitative analysis of GPX4, SLC7A11, and COX2 in the tumours excised from nude mice of different groups. Data are presented as mean ± SD; ^∗^*P* < 0.05 and ^∗∗^*P* < 0.01.

We further performed *in vivo* experiments, demonstrating that the weight and volume of tumours derived from CCSCs were decreased in the VD and sh-SLC7A11 groups. The tumour weight and volume were increased in tumours mediated by a high SLC7A11 expression, and high SLC7A11 expression alleviated the reduction in tumour weight and volume mediated by VD (Figures 7(a)–7(d)). The proliferation of CCSCs, contents of Cys and GSH, and expression levels of ferroptosis markers *in vivo* were consistent with the results *in vitro*, as SLC7A11 overexpression rescued the VD-induced ferroptosis. Knockdown of SLC7A11 significantly enhanced the effects of VD on Cys and GSH levels, ROS levels, and the levels of ferroptosis markers, such as SLC7A11 and COX2 (Figures 7(e)–7(i), Additional file 5). These results demonstrated that VD induced the ferroptosis of CCSCs by downregulating SLC7A11.

## 4. Discussion

Chemoresistance and treatment failure are the main reasons for the poor prognosis of CRC. Increased expression of CCSC biomarkers has been linked to poor prognosis in CRC patients. CCSCs play significant roles in tumour resistance to therapy, tumour metastasis, and tumour recurrence and have provided new avenues for treating CRC, which require further investigation [22]. Hence, effective targeting of this population of cells would be helpful in developing therapeutics for CRC.

Since CCSCs can form spheroids, we isolated the stem cell suspension, including cell spheroids, in DMEM/F-12. Because CD44 and CD133 are the major markers to differentiate CCSCs, we detected cell spheroids using flow cytometry. Results showed that the percentages of CD133^+^ and CD44^+^ cells in the stem cell suspension were 71.53% and 58.88%, respectively. CCSCs express many of the stemness transcription factors such as Sox2, Nanog, and Oct4, so we detected the expression levels of Sox2, Nanog, and Oct4 for further identification. Results showed that the expression levels of stemness markers were increased in the stem cell suspension, which was similar to the results of a previous study [23]. Hence, we isolated the stem cell suspension as CCSCs for the subsequent experiments.

Although VD displays an array of potential protective effects against CRC by acting on carcinoma cells, immune cells, cancer-associated fibroblasts, and probably the gut microbiota, the effect of VD on CCSCs is still unknown [24]. Our study showed that VD significantly reduced the size and number of tumour spheroids formed by CCSCs. The inhibitory effect of VD on CCSC proliferation was evaluated through the CCK-8 assay; the proliferation of CCSCs in the VD group (100 nM) was found to be significantly reduced. Furthermore, the *in vivo* experiments showed that VD treatment significantly reduced the weight and volume of tumours derived from CCSCs. These results indicate that VD might effectively inhibit the proliferation of CCSCs *in vitro* and *in vivo*.

Various mechanisms, such as the special microenvironment, ATP-binding cassette drug transporters, and several signaling pathways, have been proposed to describe the role of CCSCs in promoting chemoresistance [25]. Despite several molecular approaches used to target CCSCs, such as targeting the microenvironment, inhibiting drug efflux pumps, or modulating signaling pathways, challenges remain because the existing treatments are not yet optimum or are not very specific to CCSCs [26]. Hence, it is crucial to conduct further research on other ways to target these cells. In this study, when the CCSCs were treated with 1 *μ*M oxaliplatin and 100 nM VD, we found that VD treatment improved the drug sensitivity of oxaliplatin in CCSCs. These results indicate that VD might promote chemosensitivity in CRC by targeting CCSCs.

Ferroptosis, a form of nonapoptotic cell death caused by ROS accumulation due to iron-dependent oxidative damage, has been reported as a successful strategy against CCSCs [27]. Compared with their parental counterparts, CCSCs have certain characteristics that render them more sensitive to ferroptosis [8]. The accumulation of lethal ROS and reduced number of or vanished mitochondrial cristae are the characteristics of ferroptosis [28, 29]. Our results revealed ROS accumulation and disappearance of mitochondria when the CCSCs were treated with 100 nM VD; this suggested that CCSCs encountered ferroptosis. GPX4 is the enzyme responsible for peroxide detoxification in the cell and is the main protector of cells from ferroptosis. Decreased activity and inhibition of this enzyme were observed to drive cells into death in a ferroptotic manner [25]. System xc- is a cystine/glutamate antiporter, and the inhibition of its activity by erastin in cancer cells triggers ferroptosis; SLC7A11 is a key component of system xc- [10]. Several lines of investigation have identified prostaglandin-endoperoxide synthase 2 (PTGS2) and its gene product COX2 as downstream molecular markers of ferroptosis; furthermore, increased expression of the *PTGS2* gene and its encoded protein COX2 represents ferroptosis onset [30]. Our results revealed the decreased mRNA expression of *SLC7A11* and *GPX4* and the increased mRNA expression of *COX2* in the CCSCs treated with 100 nM VD. Overall, these results suggested that VD could induce cell death via ferroptosis in CCSCs.

The cystine/glutamate antiporter systems xc- and GPX4 play important roles in ferroptosis [10]. System xc- consists of SLC7A11 and solute carrier family 3 member 2 and imports cystine into the cell [31, 32]. Cys is one of the substrates used to synthesise GSH; it is derived from cystine decomposition. GSH is a momentous intracellular antioxidant that can protect cells from oxidative damage by acting as a reducing substrate of GPX4 to decrease ROS accumulation [33]. Our results showed that the contents of Cys and GSH in CCSCs were significantly reduced in the VD group (100 nM). This finding indicated that the inhibition of system xc- and GPX4 resulted in ROS-mediated ferroptosis by leading to the depletion of intracellular Cys levels and significantly delaying GSH synthesis [34]. These results are consistent with a previous observation that the xc- inhibitor sulfasalazine sensitises colorectal cancer cells to cisplatin via a GSH-dependent mechanism [35].

Previous studies have reported that VDR can regulate ferroptosis via GPX4 in cognitive dysfunction and acute kidney injury [36, 37]. In the present study, VD treatment reduced Cys and GSH levels and the mRNA expression levels of *SLC7A11* and *GPX4* in CCSCs. We obtained 25 colon cancer-related ferroptosis genes by the intersection of 1587 DEGs and the ferroptosis-related gene dataset. SLC7A11 was included in the 25 genes, and its expression was increased while that of GPX4 was not. Hence, we hypothesised that VD treatment might inhibit the expression of SLC7A11 and deplete the intracellular levels of Cys and GSH to induce ferroptosis.

SLC7A11 is the core target for ferroptosis regulation; the overexpression of which dictates downregulated sensitivity to ferroptosis in cancer cells. The SLC7A11 expression in colon adenocarcinoma is positively associated with microsatellite instability and immunotherapeutic response [38]. In our study, knockdown of SLC7A11 significantly inhibited the proliferation of CCSCs, decreased Cys and GSH levels, and increased ROS levels. At the same time, knockdown of SLC7A11 significantly increased the protein and mRNA expression levels of COX2 while reducing GPX4 levels. Transmission electron microscopy results demonstrated that the mitochondrial membrane was thickened and mitochondria were narrowed in cells in the sh-SLC7A11 group. This indicates that SLC7A11 plays a critical role in the ferroptosis of CCSCs; these results are consistent with those of previous studies [10, 14].

A previous study reported that inhibition of ferroptosis attenuated aortic calcification in VD3-overloaded mice as an animal model of chronic kidney disease. Mechanistically, high levels of VD downregulated SLC7A11, reduced GSH content in vascular smooth muscle cells (VSMCs), and initiated ferroptotic cell death, leading to the calcification of VSMCs [39]. In our study investigating the relationship between VD and SLC7A11, SLC7A11 was overexpressed to determine its role in VD-induced ferroptosis in CCSCs. Additionally, we determined the effect of SLC7A11 on Cys, GSH, and cell proliferation levels by overexpressing SLC7A11 when CCSCs were treated with VD. Our results showed that SLC7A11 overexpression rescued the Cys and GSH levels reduced by VD. Furthermore, a high SLC7A11 expression rescues the decrease in tumour weight and volume mediated by VD *in vivo*. In summary, SLC7A11 overexpression rescued the viability of CCSCs diminished by VD *in vivo* and *in vitro*.

In addition, we found that knockdown of SLC7A11 significantly enhanced the effects of VD on the size and number of tumour spheroids and the levels of Cys, GSH, ROS, and ferroptosis markers, such as SLC7A11 and COX2. However, after SLC7A11 silencing, VD had no significant promoting effect on ferroptosis in CCSCs; this suggests that the effects of VD on ferroptosis in CCSCs could mainly be based on the SLC7A11 pathway. Our study may provide a novel mechanism by which VD induces ferroptosis through SLC7A11 downregulation. However, elucidating the specific molecular mechanism underlying the relationship between VD and the SLC7A11 pathway requires further research.

## 5. Conclusions

This study discovered the effects of VD on ferroptosis in CCSCs *in vitro* and *in vivo*, unravelling how VD induces ferroptosis via SLC7A11 downregulation. Our results provide a new therapeutic reference for the use of VD in the treatment of CRC and offer new insights into its effect on ferroptosis.

## Data Availability

All the data can be obtained from the corresponding author.

## Supplementary Materials

Supplementary 1 Additional file 1: percentage of dead cells detected using flow cytometry when colorectal cancer stem cells (CCSCs) were treated with 1 *μ*M oxaliplatin and 100 nM vitamin D (VD). PE-A and FITC-A are axis labels, and P1 is the gate label. Q2-UL, Q2-UR, Q2-LL, and Q2-LR are quartile labels. Dead cells were stained with PI, and their percentages were revealed in the Q2-UR label when detected using flow cytometry.

Supplementary 2 Additional file 2: levels of ROS in the CCSCs treated with different concentrations of VD, as detected using flow cytometry. Results are expressed as percentages. Count and FITC-A are axis labels, P1 and P2 are gate labels, and there were no quartile labels. The group of negative control was used to determine the site of P2. The count of P2 represents ROS levels.

Supplementary 3 Additional file 3: (A, B) qRT-PCR analysis of SLC7A11 expression in CCSCs after transfection with sh-SLC7A11 or OE-SLC7A11. (C) Levels of ROS in the CCSCs transfected with sh-SLC7A11 or OE-SLC7A11, as detected using flow cytometry. Results are expressed as percentages. Count and FITC-A are axis labels, P1 and P2 are gate labels, and there were no quartile labels. The group of negative control was used to determine the site of P2. The count of P2 represents ROS levels.

Supplementary 4 Additional file 4: cellular levels of ROS in CCSCs detected using flow cytometry. Results are expressed as percentages. Count and FITC-A are axis labels, P1 and P2 are gate labels, and there were no quartile labels. The negative control was used to determine the site of P2. The count of P2 represents ROS levels.

Supplementary 5 Additional file 5: cellular levels of ROS in the tumour excised from nude mice, as detected using flow cytometry. Results are expressed as percentages. Count and FITC-A are axis labels, P1 and P2 are gate labels, and there were no quartile labels. The negative control was used to determine the site of P2. The count of P2 represents ROS levels.
