# Supplementary figures and images for: Vitamin D Promotes Ferroptosis in Colorectal Cancer Stem Cells via SLC7A11 Downregulation
Source: Oxid Med Cell Longev. 2023 Feb 16;2023:4772134. doi: 10.1155/2023/4772134 (PMC9950793; doi:10.1155/2023/4772134)

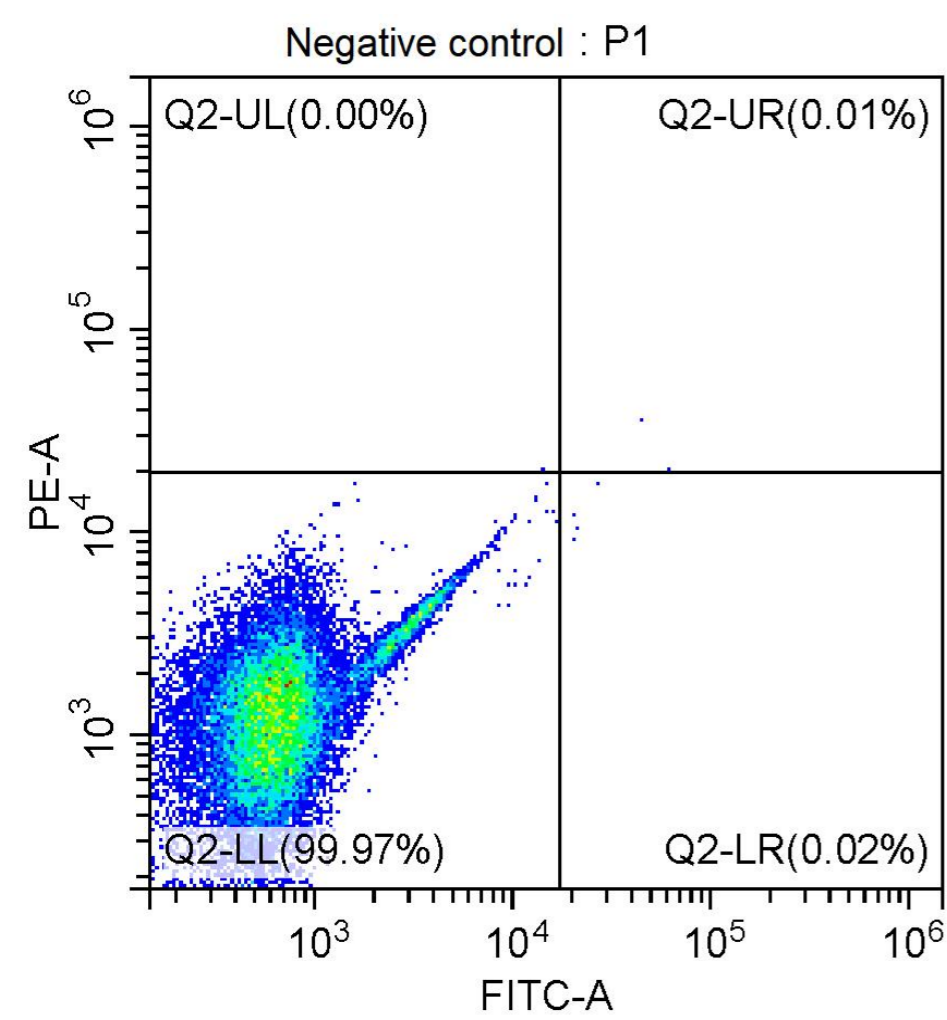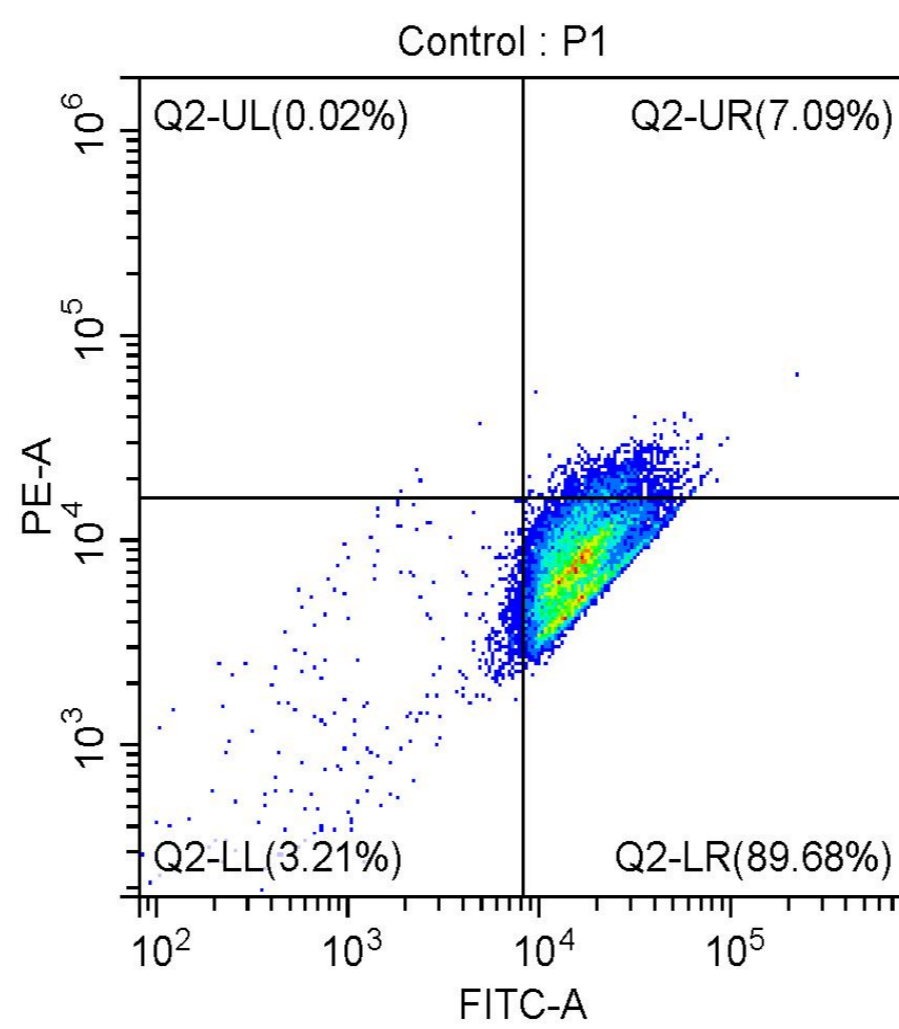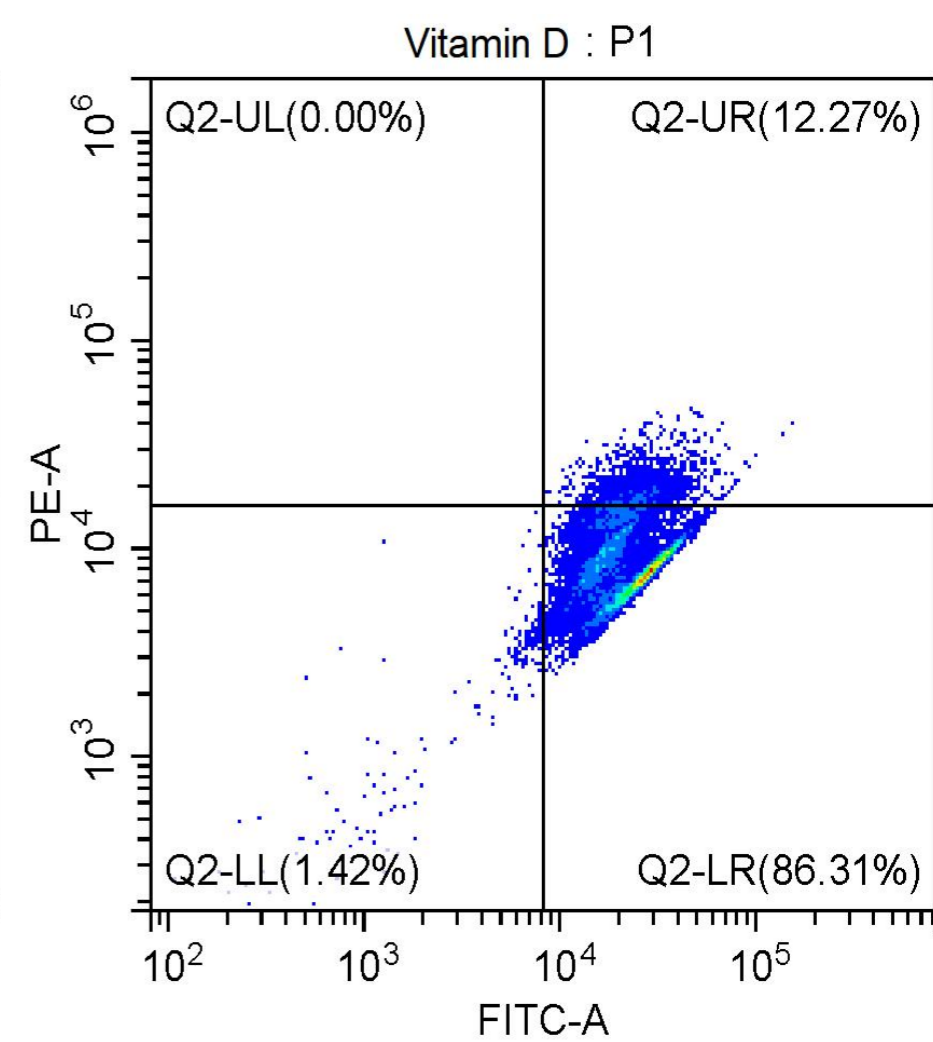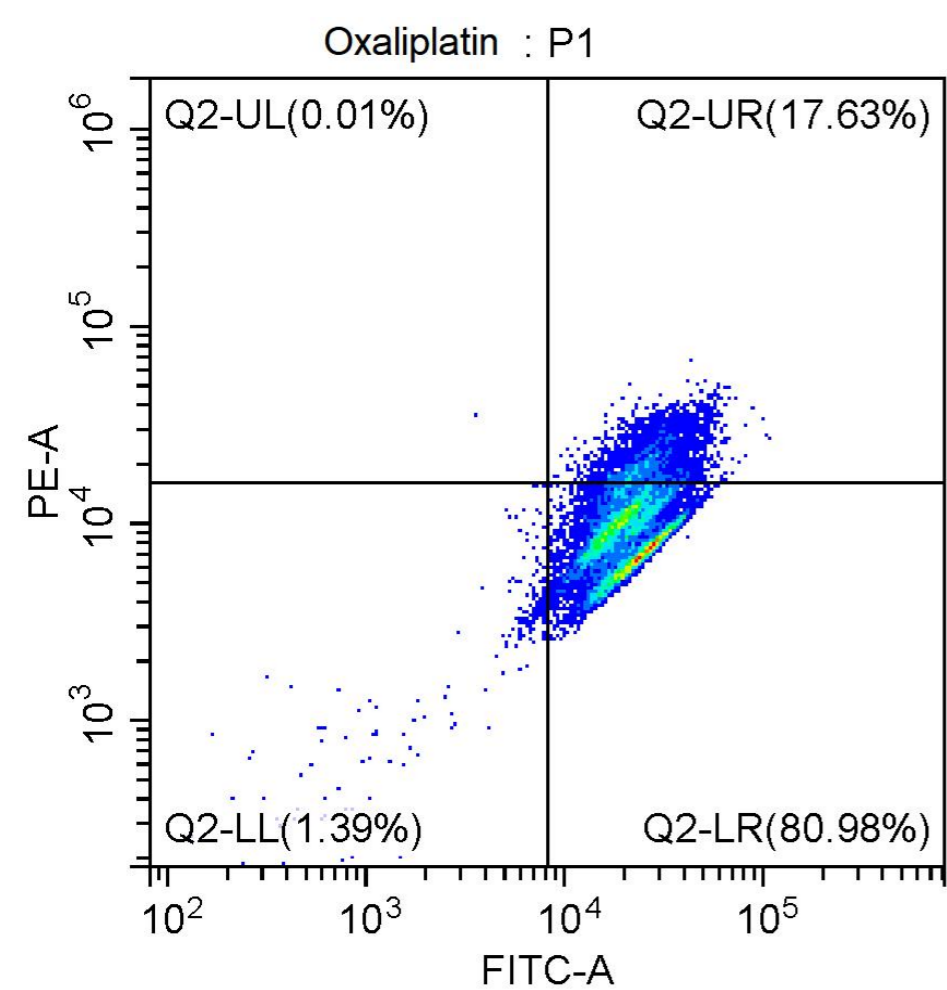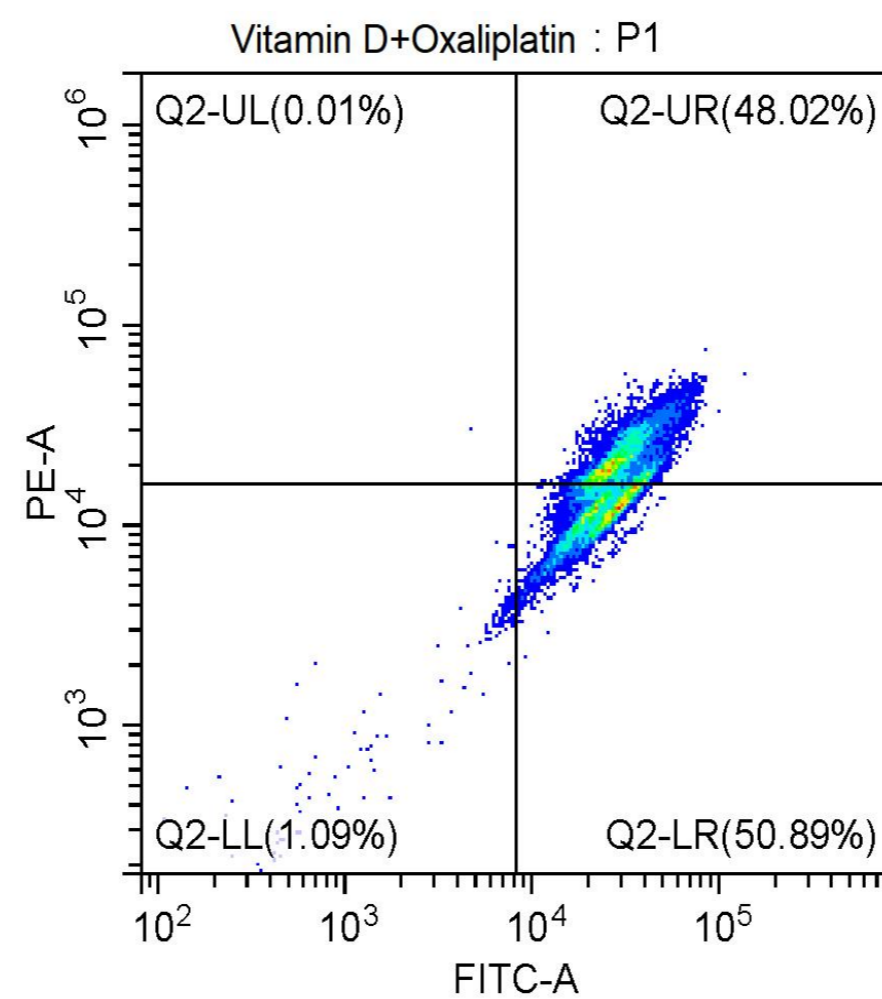

1 $\mu$ m

Supplement: Supplementary 1 — Additional file 1: percentage of dead cells detected using flow cytometry when colorectal cancer stem cells (CCSCs) were treated with 1 μM oxaliplatin and 100 nM vitamin D (VD). PE-A and FITC-A are axis labels, and P1 is the gate label. Q2-UL, Q2-UR, Q2-LL, and Q2-LR are quartile labels. Dead cells were stained with PI, and their percentages were revealed in the Q2-UR label when detected using flow cytometry. [file 4772134.f1.pdf]

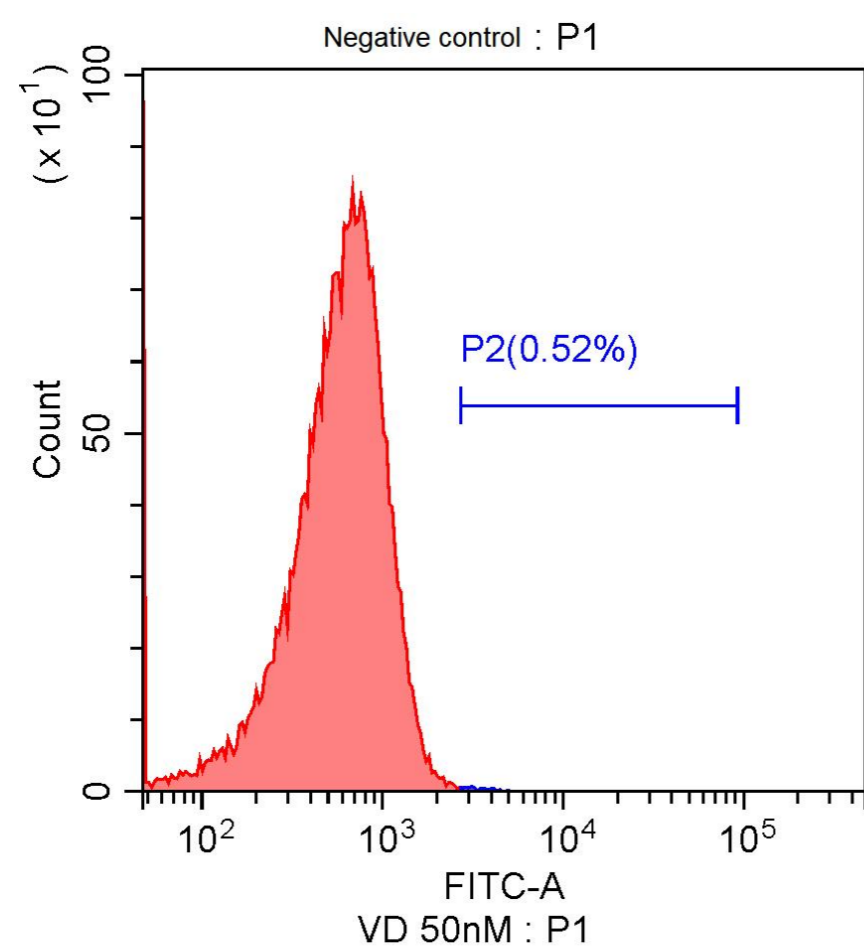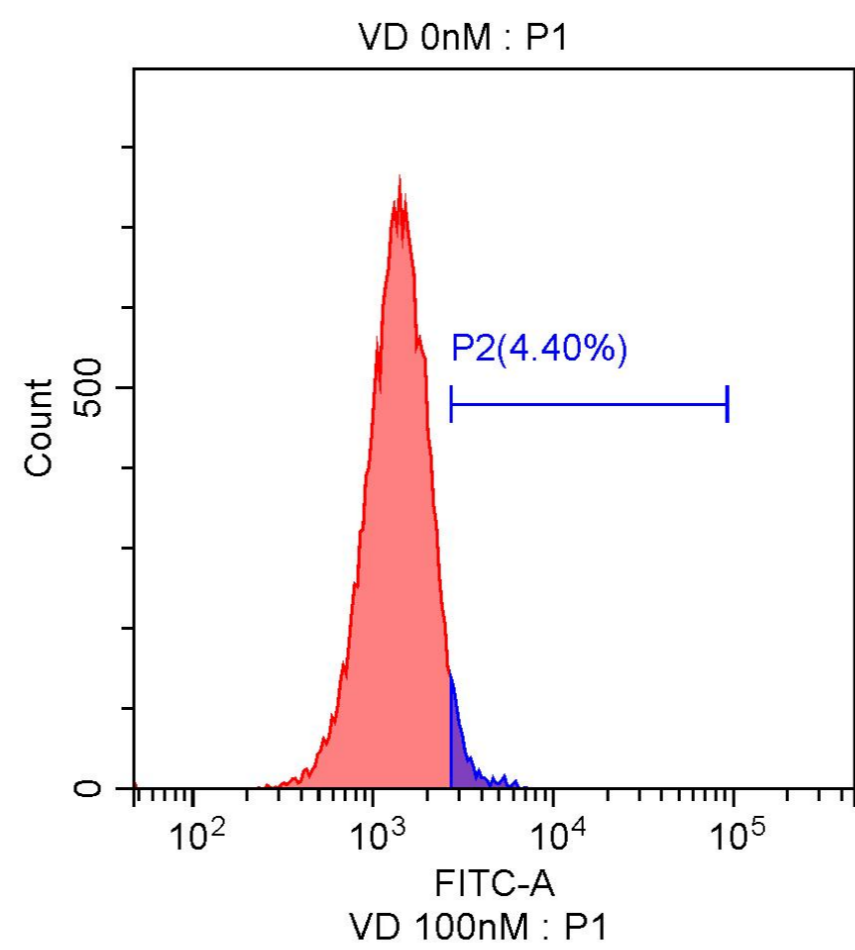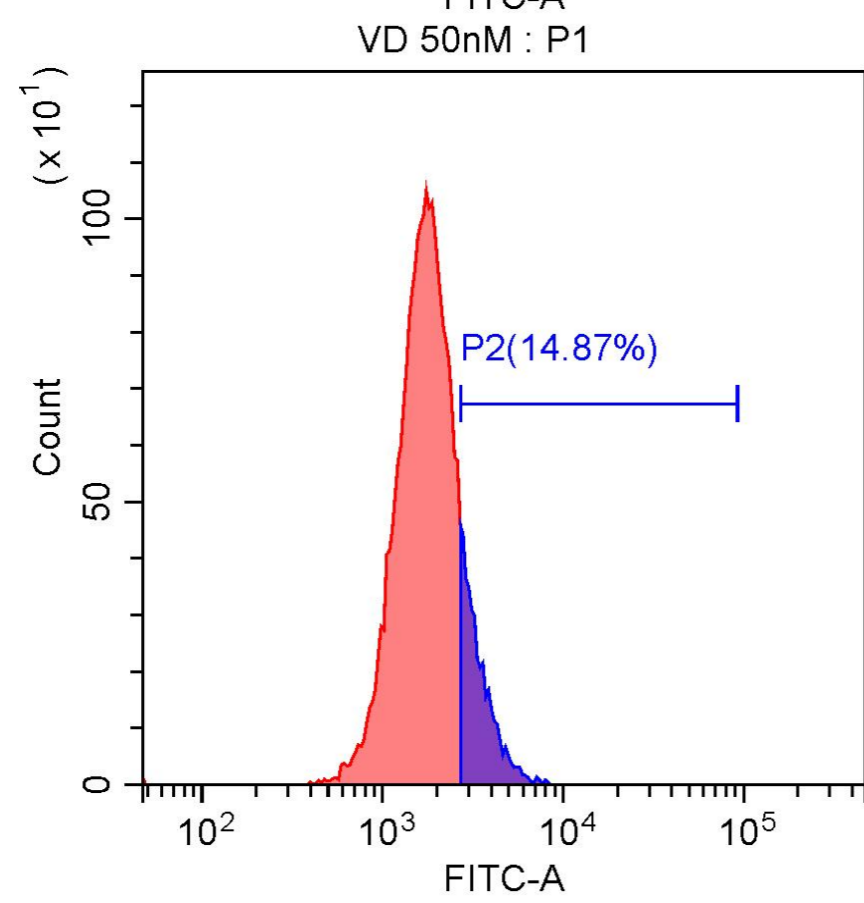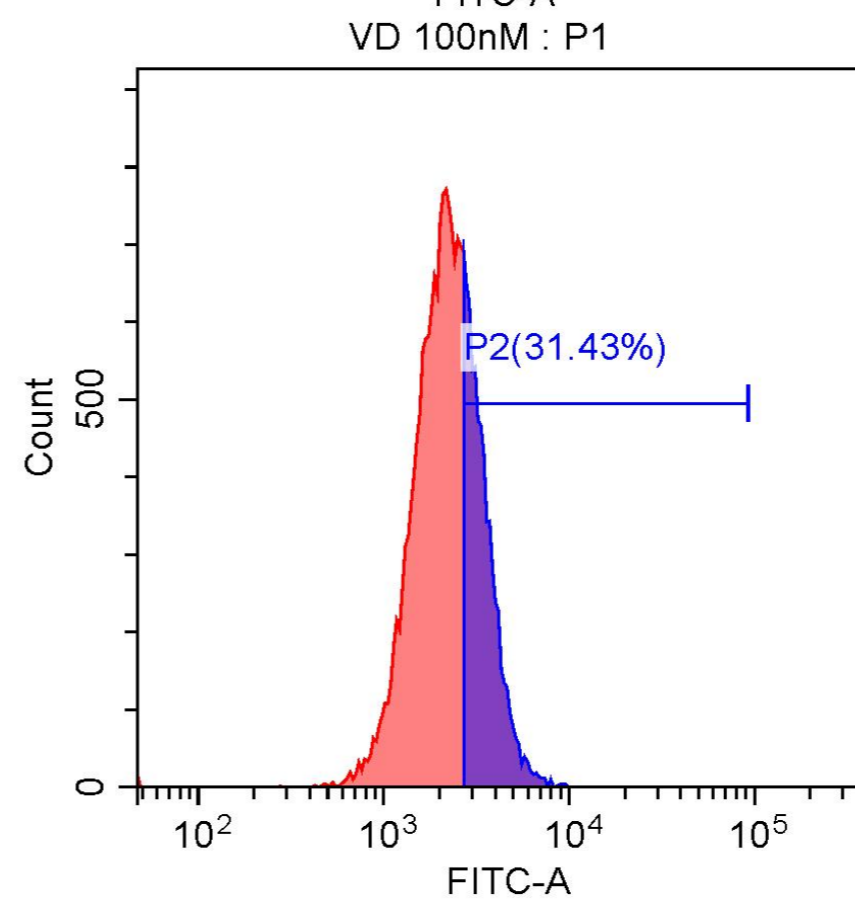

Supplement: Supplementary 2 — Additional file 2: levels of ROS in the CCSCs treated with different concentrations of VD, as detected using flow cytometry. Results are expressed as percentages. Count and FITC-A are axis labels, P1 and P2 are gate labels, and there were no quartile labels. The group of negative control was used to determine the site of P2. The count of P2 represents ROS levels. [file 4772134.f2.pdf]

**A**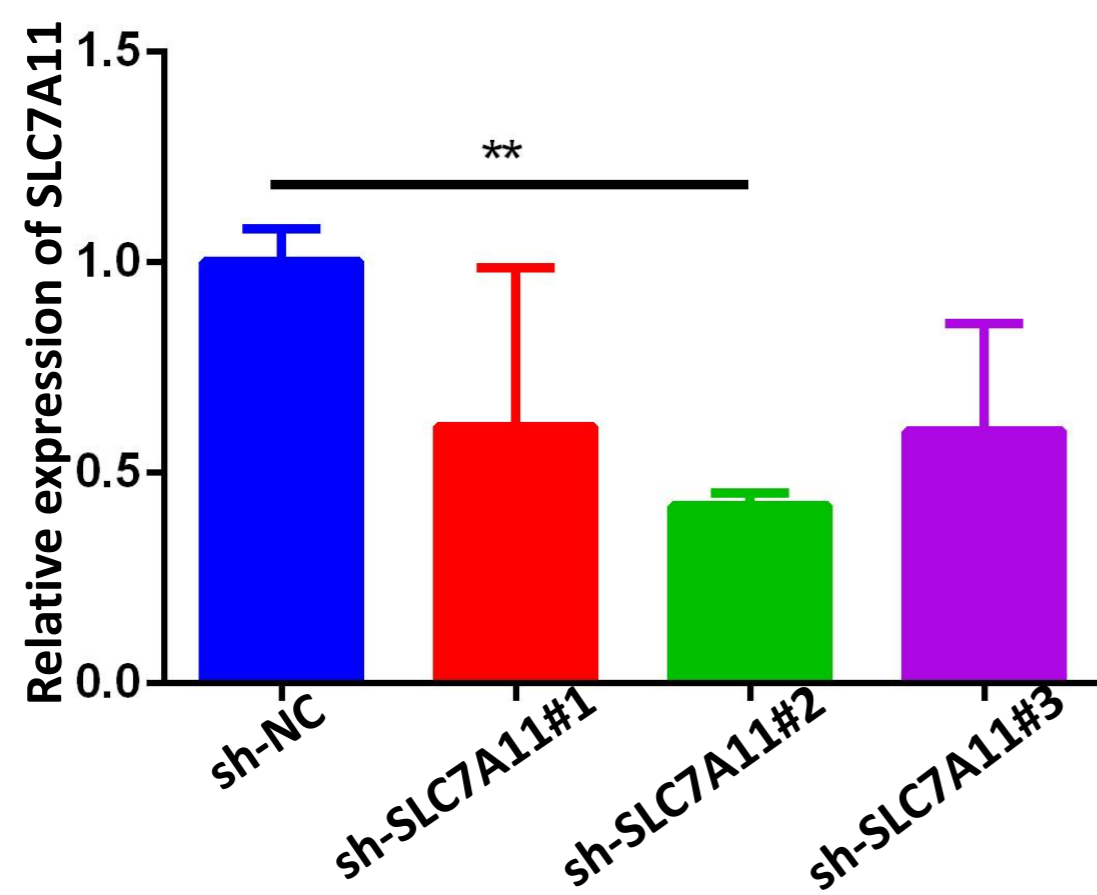**B**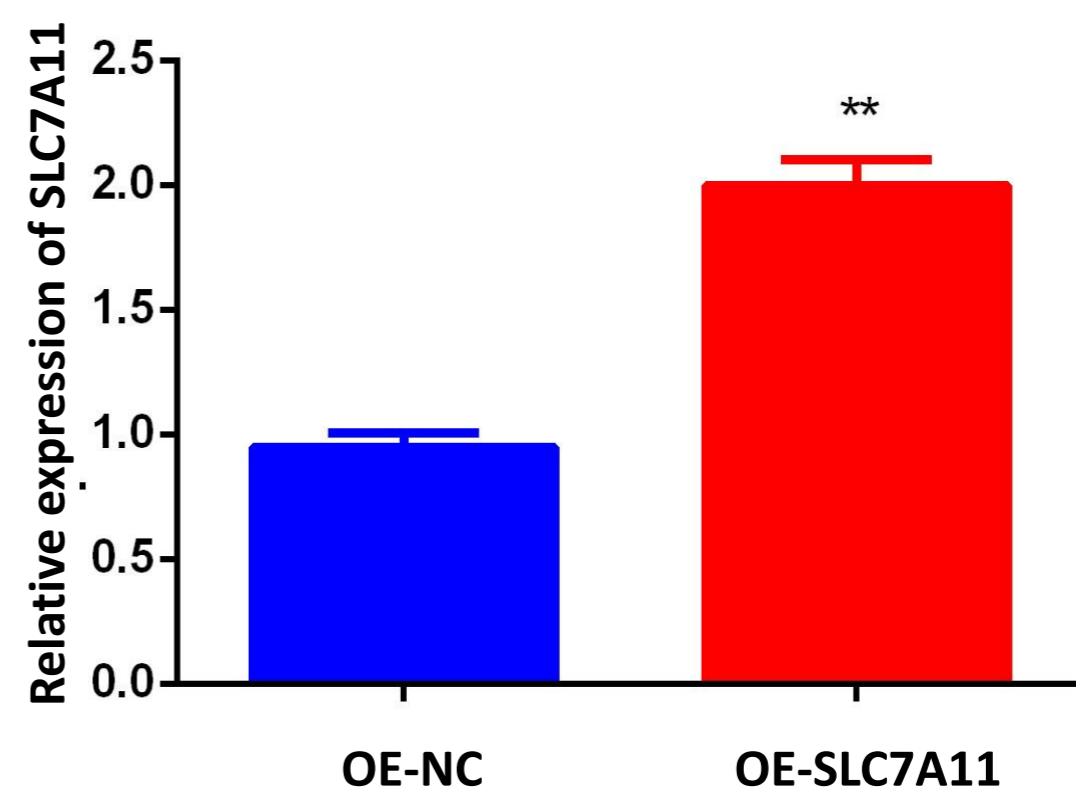**C**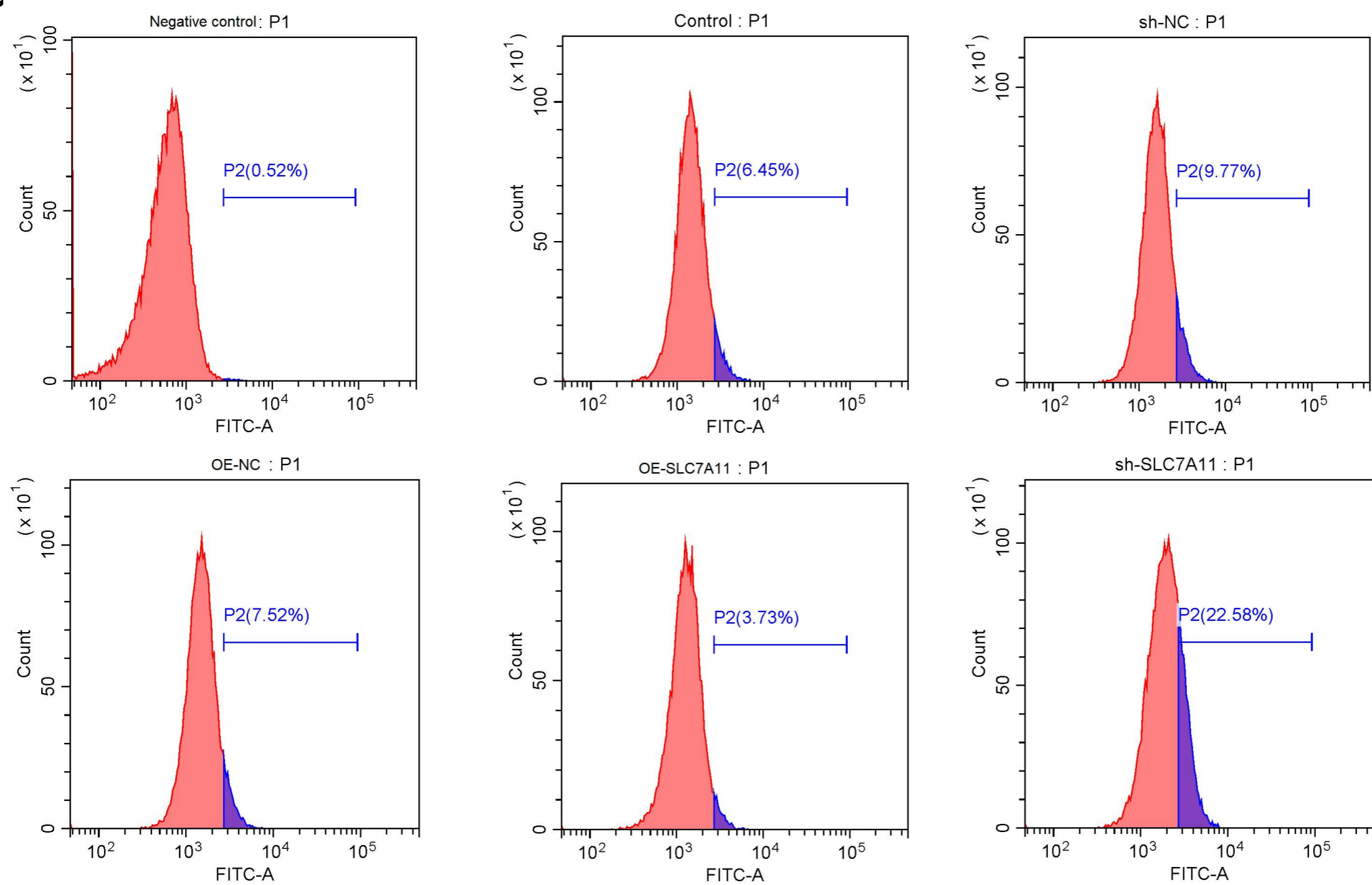

Supplement: Supplementary 3 — Additional file 3: (A, B) qRT-PCR analysis of SLC7A11 expression in CCSCs after transfection with sh-SLC7A11 or OE-SLC7A11. (C) Levels of ROS in the CCSCs transfected with sh-SLC7A11 or OE-SLC7A11, as detected using flow cytometry. Results are expressed as percentages. Count and FITC-A are axis labels, P1 and P2 are gate labels, and there were no quartile labels. The group of negative control was used to determine the site of P2. The count of P2 represents ROS levels. [file 4772134.f3.pdf]

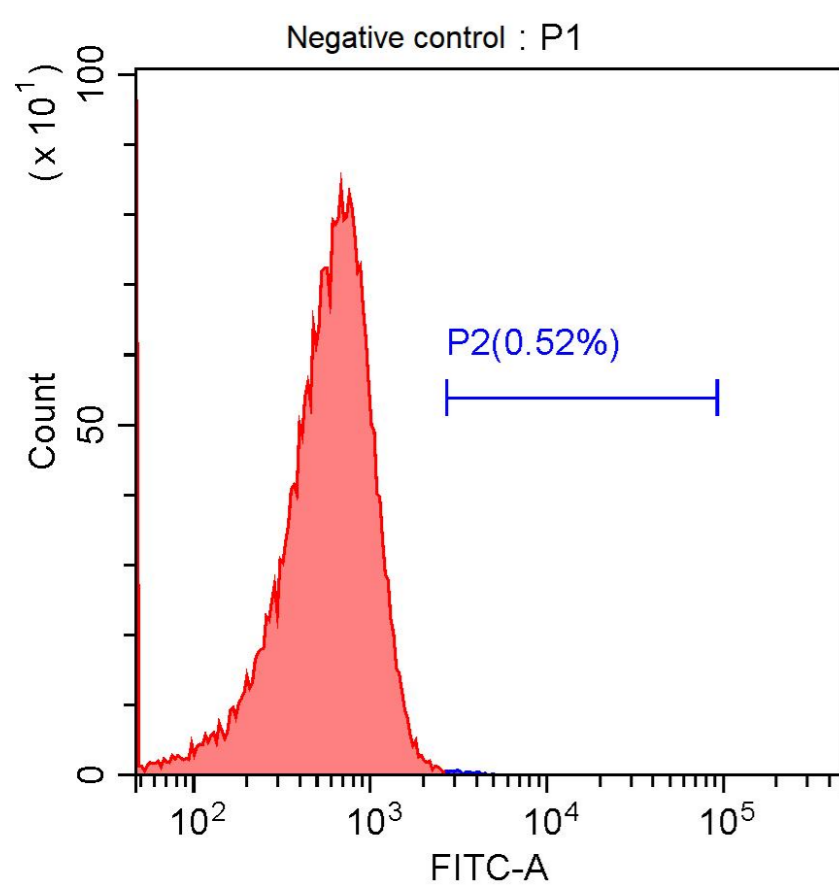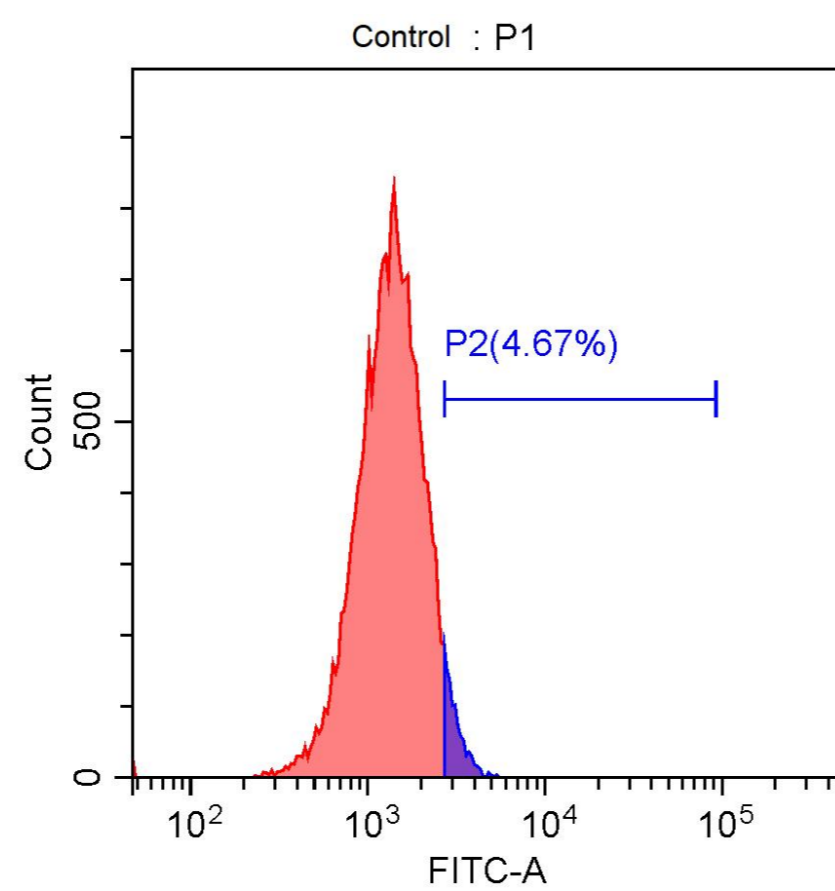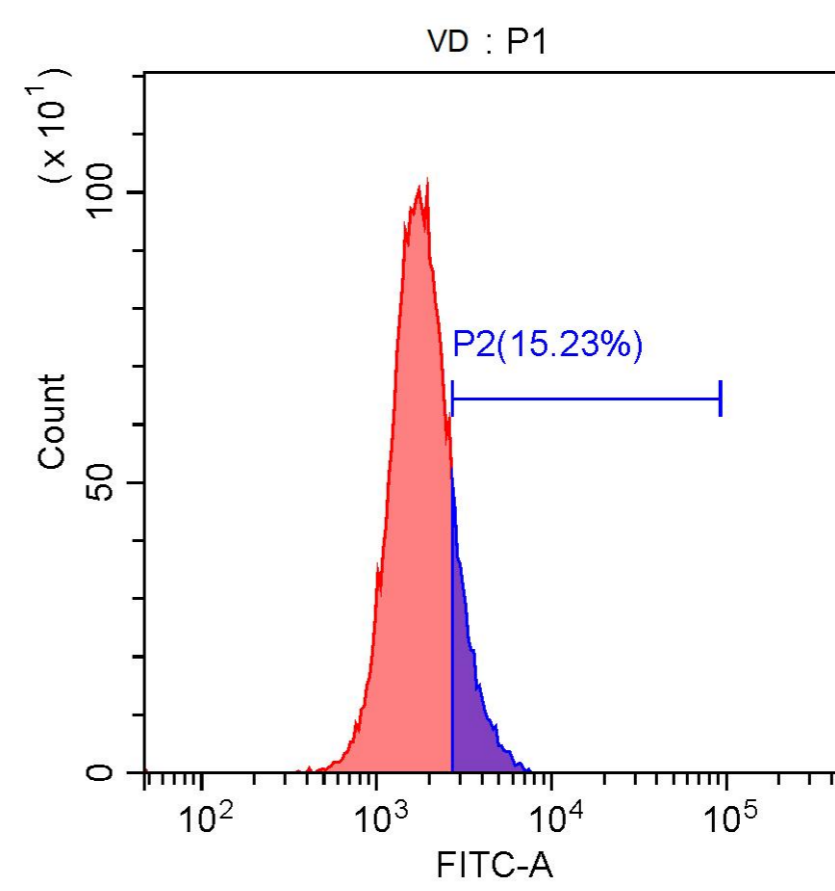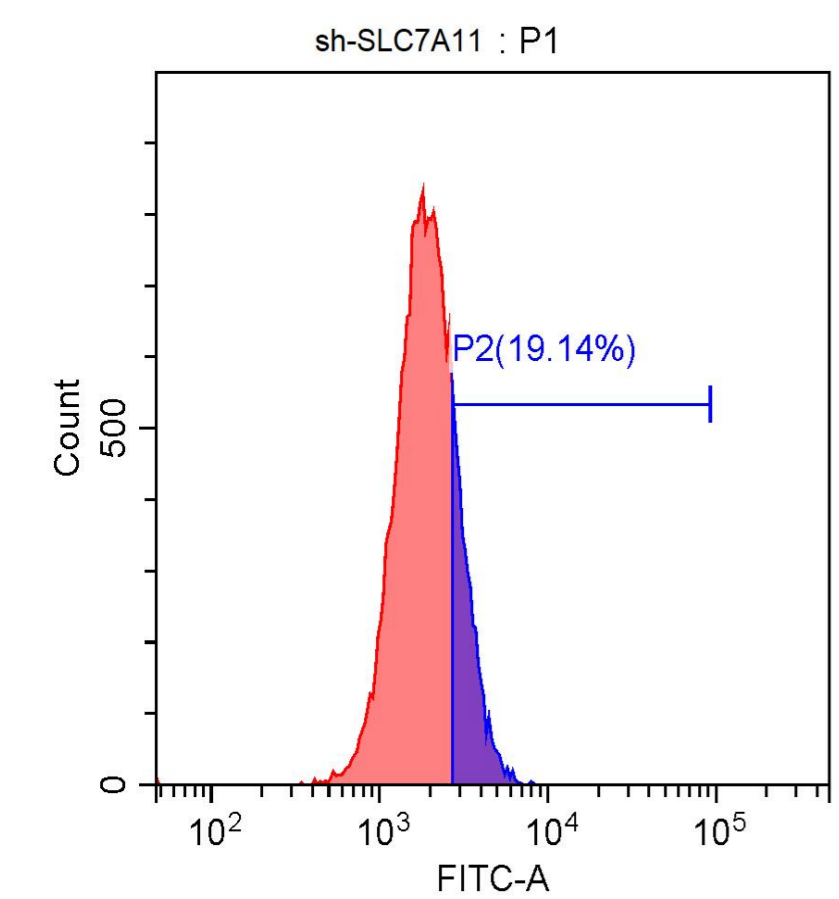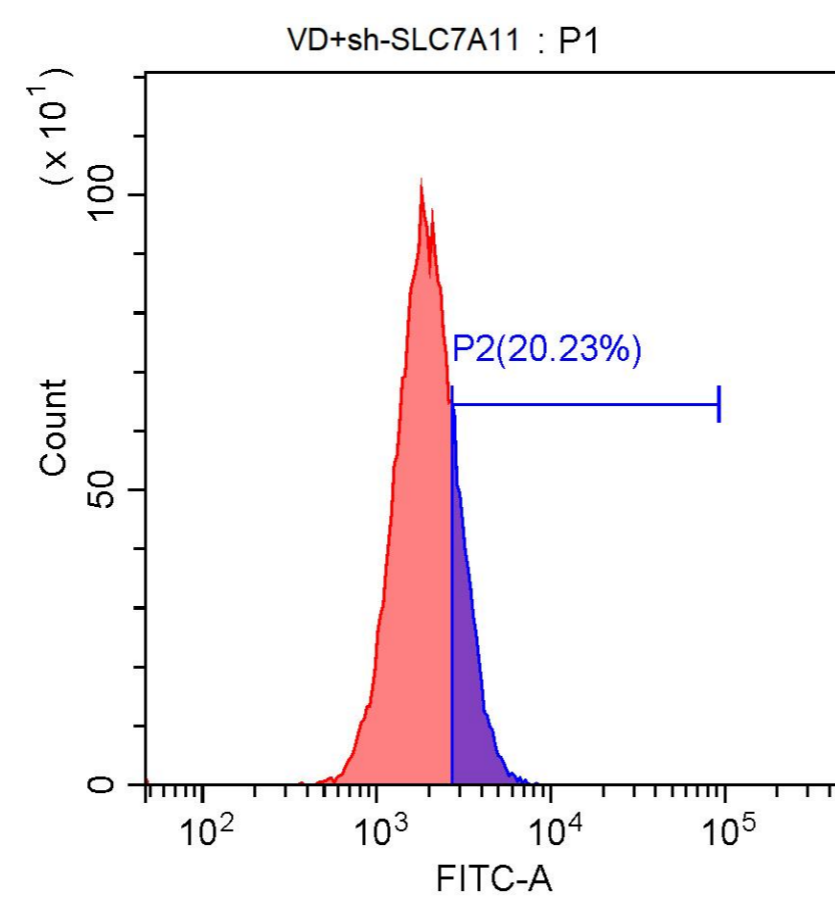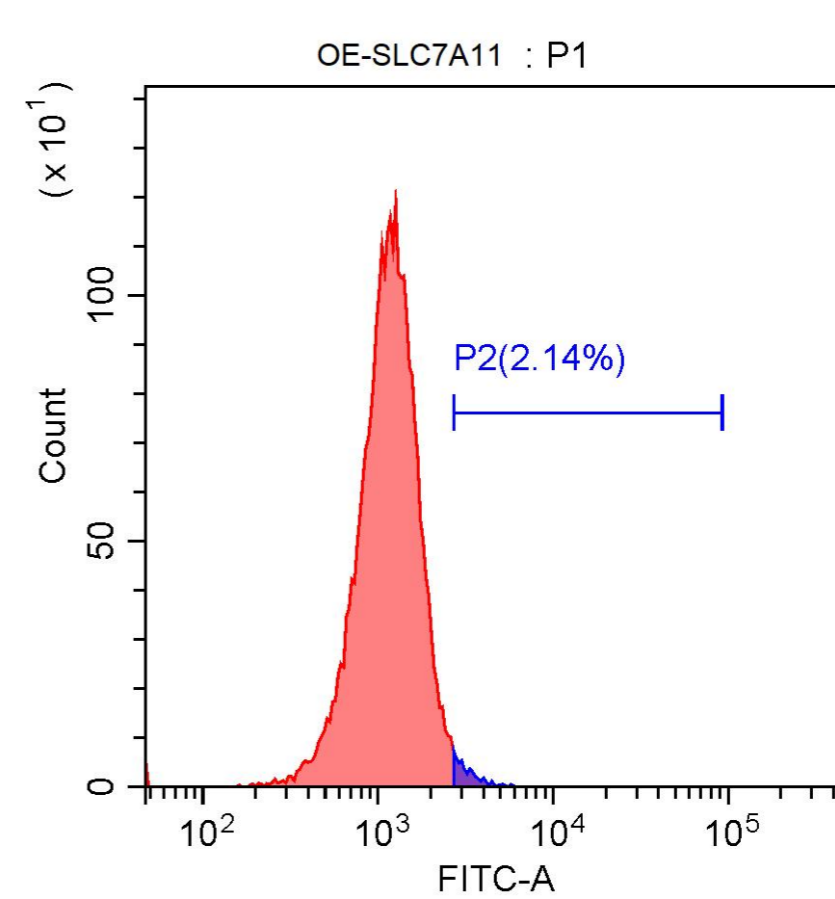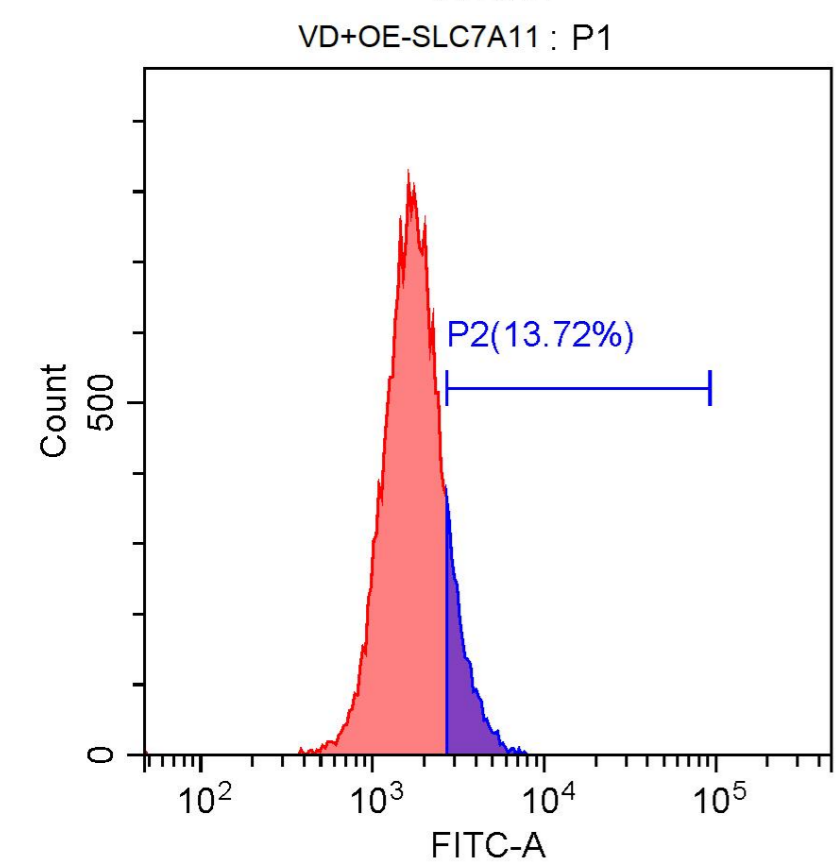

Supplement: Supplementary 4 — Additional file 4: cellular levels of ROS in CCSCs detected using flow cytometry. Results are expressed as percentages. Count and FITC-A are axis labels, P1 and P2 are gate labels, and there were no quartile labels. The negative control was used to determine the site of P2. The count of P2 represents ROS levels. [file 4772134.f4.pdf]

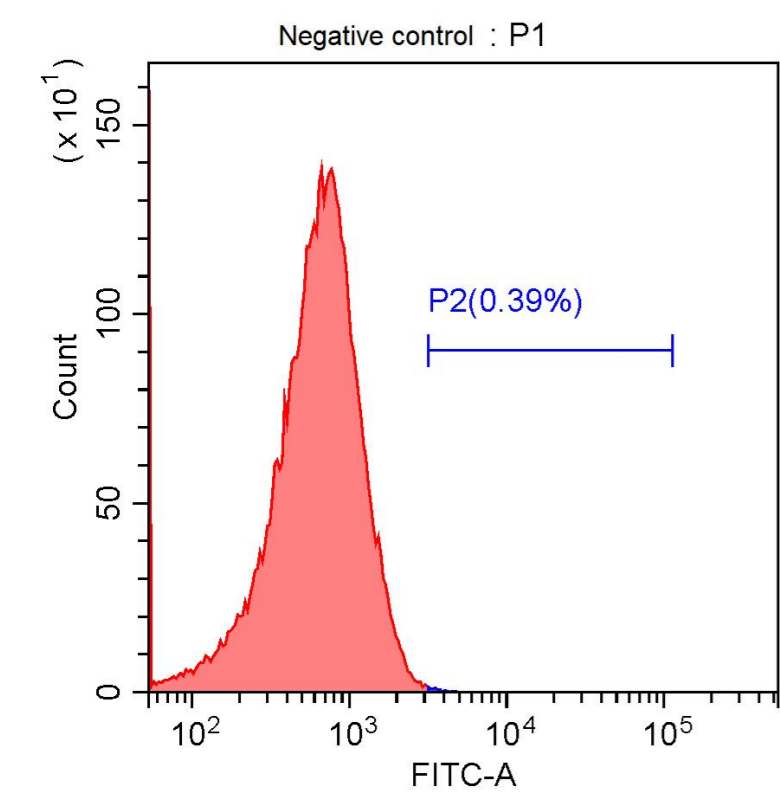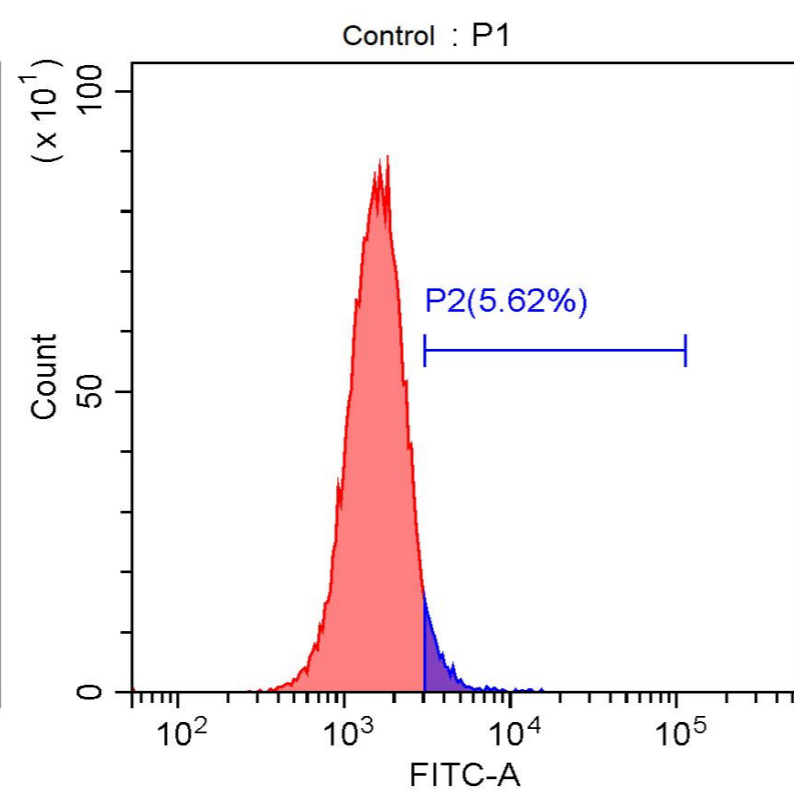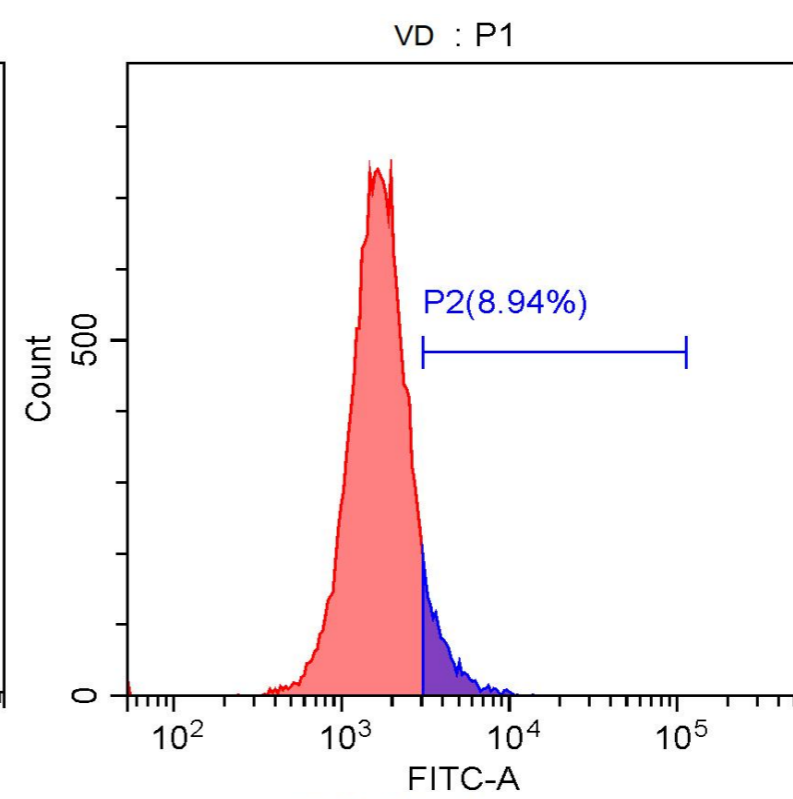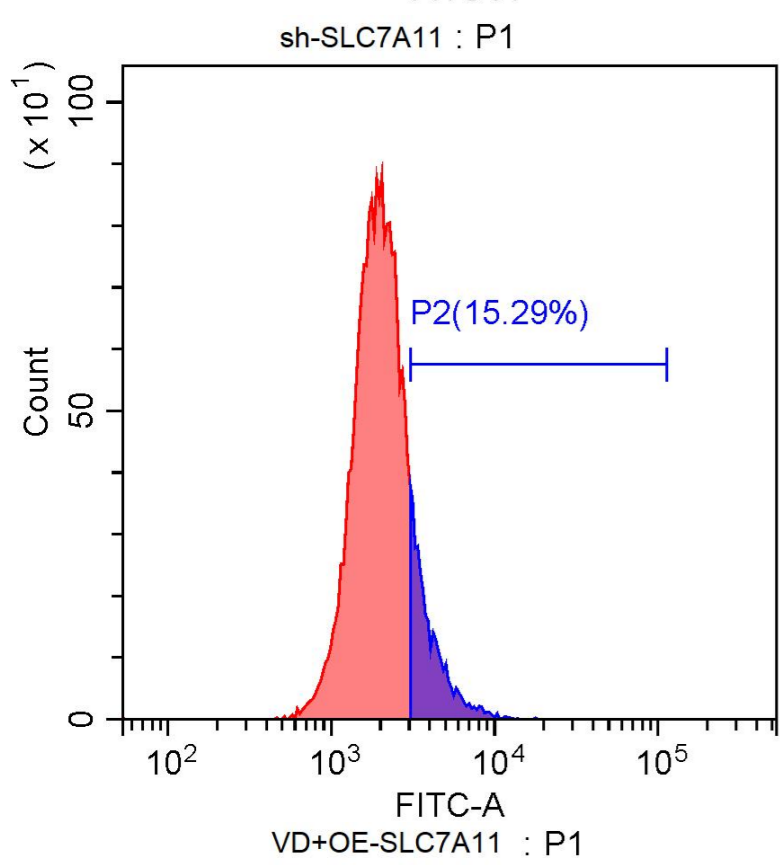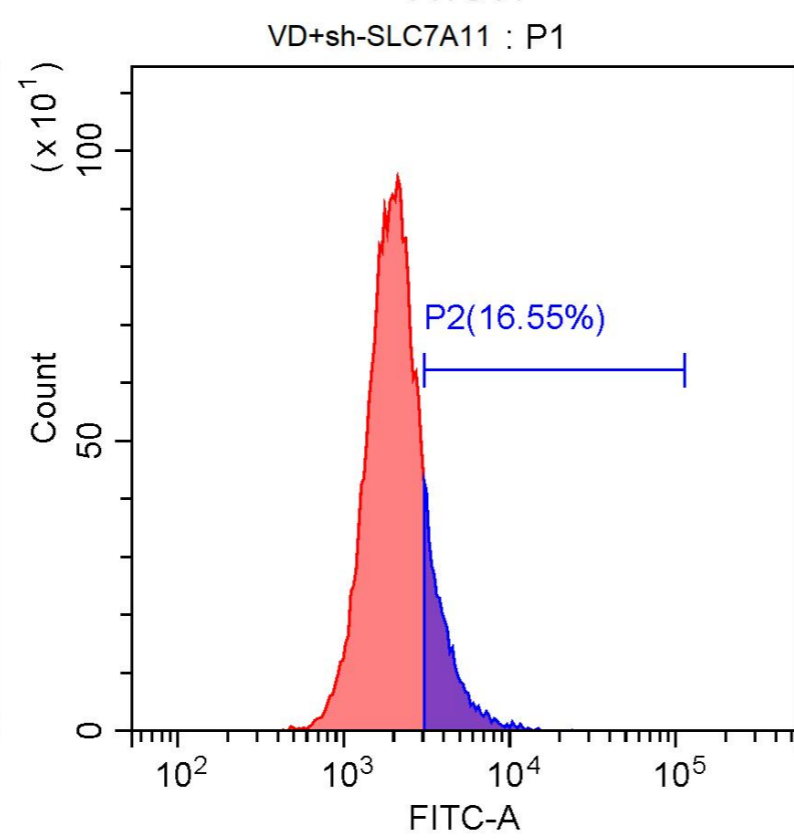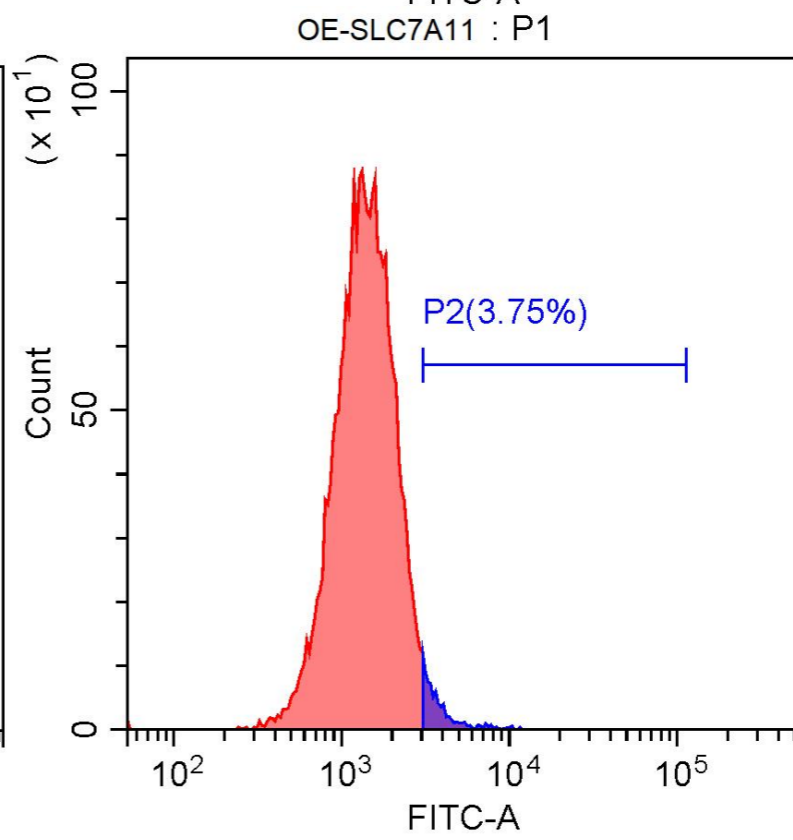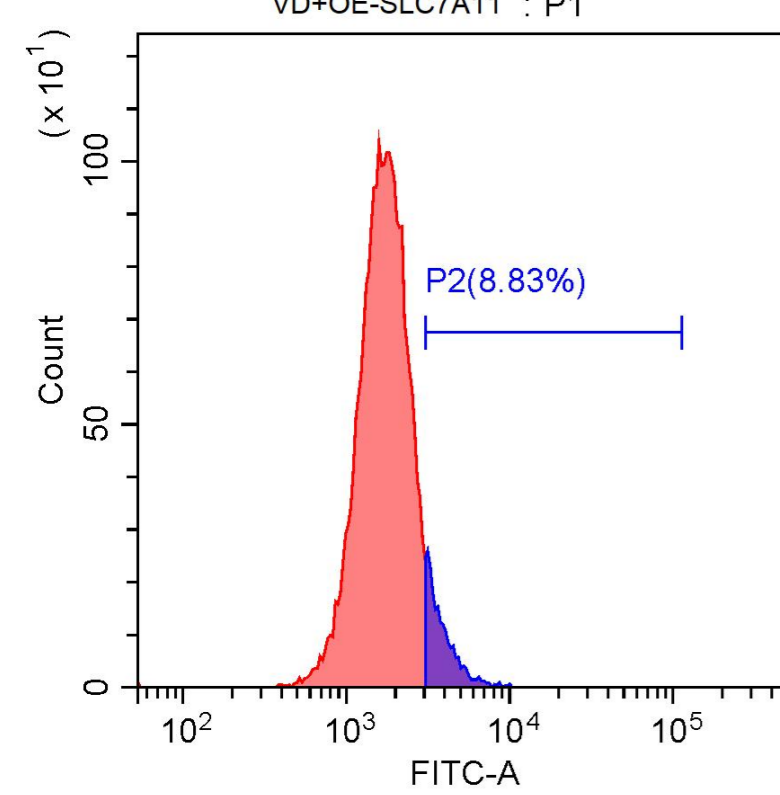

Supplement: Supplementary 5 — Additional file 5: cellular levels of ROS in the tumour excised from nude mice, as detected using flow cytometry. Results are expressed as percentages. Count and FITC-A are axis labels, P1 and P2 are gate labels, and there were no quartile labels. The negative control was used to determine the site of P2. The count of P2 represents ROS levels. [file 4772134.f5.pdf]
